# Revision of ***Sternaspis*** Otto, 1821 (Polychaeta, Sternaspidae)

**DOI:** 10.3897/zookeys.286.4438

**Published:** 2013-04-08

**Authors:** Kelly Sendall, Sergio I. Salazar-Vallejo

**Affiliations:** 1 Royal British Columbia Museum, Natural History Section, Victoria, Canada; 2 El Colegio de la Frontera Sur, CONACYT, Departamento de Ecología Acuática, Chetumal, México

**Keywords:** Widespread species, taxonomy, systematic, Annelida, Echiurida, ventro-caudal shield

## Abstract

To the memory of William Ronald Sendall

Sternaspid polychaetes are common and often abundant in soft bottoms in the world oceans. Some authors suggest that only one species should be recognized, whereas others regard a few species as widely distributed in many seas and variable depths from the low intertidal to about 4400 m. There are some problems with species delineation and the distinctive ventro-caudal shield has been disregarded or barely used for identifying species. In order to clarify these issues, the ventral shield is evaluated in specimens from the same locality and its diagnostic potential is confirmed. On this basis, a revision of *Sternaspis* Otto, 1821 (Polychaeta: Sternaspidae) is presented based upon type materials, or material collected from type localities. The sternaspid body, introvert hooks and shield show three distinct patterns, two genera have seven abdominal segments and tapered introvert hooks, and one genus has eight abdominal segments and spatulate introvert hooks. The ventro-caudal shield has three different patterns: stiff with ribs, and sometimes concentric lines, stiff with feebly-defined ribs but no concentric lines, and soft with firmly adhered sediment particles. *Sternaspis* is restricted to include species with seven abdominal segments, falcate introvert hooks, and stiff shields, often exhibiting radial ribs, concentric lines or both. *Sternaspis* includes, besides the type species, *Sternaspis thalassemoides* Otto, 1821 from the Mediterranean Sea, *Sternaspis affinis* Stimpson, 1864 from the Northeastern Pacific, *Sternaspis africana* Augener, 1918, **stat. n.** from Western Africa, *Sternaspis andamanensis* **sp. n.** from the Andaman Sea, *Sternaspis costata* von Marenzeller, 1879 from Japan, *Sternaspis fossor* Stimpson, 1853 from the Northwestern Atlantic, *Sternaspis islandica* Malmgren, 1867 from Iceland, *Sternaspis maior* Chamberlin, 1919 from the Gulf of California, *Sternaspis princeps* Selenka, 1885 from New Zealand, *Sternaspis rietschi* Caullery, 1944 from abyssal depths around Indonesia, *Sternaspis scutata* (Ranzani, 1817) from the Mediterranean Sea, *Sternaspis spinosa* Sluiter, 1882 from Indonesia, and *Sternaspis thorsoni* **sp. n.** from the Iranian Gulf. Two genera are newly proposed to incorporate the remaining species: *Caulleryaspis* and *Petersenaspis*. *Caulleryaspis* **gen. n.** is defined by the presence of falcate introvert hooks, seven abdominal segments, and soft shields with sediment particles firmly adhered on them; it includes two species: *Caulleryaspis gudmundssoni* **sp. n.** from Iceland and *Caulleryaspis laevis* (Caullery, 1944) **comb. n.** from Indonesia. *Petersenaspis* **gen. n.** is defined by the presence of spatulate introvert hooks, eight abdominal segments, and stiff shields with poorly defined ribs but no concentric line; it includes *Petersenaspis capillata* (Nonato, 1966) from Brazil and *Petersenaspis palpallatoci* **sp. n.** from the Philippines. Neotypes are proposed for eight species: *Sternaspis thalassemoides*, *Sternaspis affinis*, *Sternaspis africana*, *Sternaspis costata*, *Sternaspis fossor*, *Sternaspis maior*, *Sternaspis scutata* and *Sternaspis spinosa*, to stabilize these species-group names, and a lectotype is designated for *Sternaspis laevis* which is transferred to *Caulleryaspis* **gen. n.** The geographic range of most species appears to be much smaller than previously indicated, and for some species additional material in good condition is needed to clarify their distributions. Keys to genera and to all species are also included.

## Introduction

The peculiar, peanut-shaped sternaspid polychaetes have been known since the eighteenth century because they are common in shallow water sandy bottoms. After the first observations, their body shape was regarded as resembling a squash and hence its non-Linnean name as *Mentula cucurbitacea marina* (Plancus 1760), but others call them gooseberry worms (Hartman and Reish 1950). Otto (1821) proposed *Sternaspis*, the genus name that now includes most described species, but one species had been formally described a few years before (Ranzani 1817). The name was derived from two Greek words meaning breast (*stern*, m.) and shield (*aspis*, f.) because Otto confused the body ends, whereas Ranzani had identified them correctly (Eysenhardt 1818). The diagnosis by de Blainville (1828: 500–501) repeated Otto’s confusion but corrected it in the legend for figures that were realigned for body ends, and this was later confirmed by Audouin and Milne-Edwards (1829: 82). Their colourful ventro-caudal shield has made these polychaetes easily recognized and explains the common name of ‘mud-owls’; this name is explained because the shield resembles the owl’s large eyes, whereas the body resembles the bird’s resting body shape.

Sternaspidae is a monogeneric family of polychaetes with 13 nominal species and two subspecies (Petersen 2000). However, one or two species are recorded from many different localities and they are regarded as cosmopolitans (Hartman and Reish 1950). All *Sternaspis* species are typically sub-littoral, marine, infaunal and non-selective, direct deposit-feeders. Since the first record in the literature in mid-1700 (Plancus 1760), members of this genus have been reported from all oceans of the world. Although they have been collected from depths as great as 4418 m (Kirkegaard 1983), they are more likely to be collected from depths less than 200 m (Fauchald 1977). They have been collected from a variety of substrates such as rocky sand (Hartman 1963), coarse sand, broken shell, soft mud (Treadwell 1914), and deep sea clays and muds (Rouse and Pleijel 2001). As Southern (1928) reported *Sternaspis costata* von Marenzeller, 1879 from Chilka (now Chilika) Lake, a brackish inland saltwater lagoon in the northeast Province of Orissa, India, it appears that at least one *Sternaspis* species tolerates low salinities.

The type of substrate apparently regulates how sternaspids live. In sandy bottoms, they partially bury themselves head first into the sediment with the posterior end above the sediment surface, thereby exposing the branchiae to oxygenated water (KS pers. obs.). In muds, the body of sternaspids takes on a depressed form (Dorgan et al. 2006), and they are found below the water-sediment interface. These contradictory observations will hopefully encourage future studies about their living pattern, potential speciation processes and how they defecate. Regarding the latter, old illustrations show sternaspids with a prolapsed rectum, but this cylindrical structure might actually be a caudal peduncle, like the one found in some sabellariids.

Sternaspidae include abundant or dominant species and this emphasizes the need to clarify their taxonomic status. In the Central Adriatic Sea, de Biasi and de Raineri (2006) found that *Sternaspis* is more abundant in fished bottoms than in a non-fished control sites. Harmelin-Vivien et al. (2009) noticed that in the NW Mediterranean Sea, *Sternaspis* species increased in abundance depending on the amount of the particulate organic matter load in rivers and this increases the production of common soles, *Solea solea* (Linneaus, 1758) . *Sternaspis* sp. was the most abundant species along the southwestern coast of India (Joydas and Damodaran 2009), in 30–50 m and in sandy, muddy or mixed bottoms, there were up to 1335 specimens per square metre. Likewise, in shallow water muddy bottoms in Bahia, Brazil an apparently undescribed species was the most abundant benthic species (Pires-Vanin et al. 2011); a different species, identified as *Sternaspis scutata*, was the most abundant in Jiaozhou Bay, China (Wang et al. 2006), and a similar condition was recorded for southern Chile (Rozbaczylo et al. 2006). The study of these materials can help improve our knowledge about species variation and to facilitate their recognition as distinct species.

Studies on the reproduction and development of sternaspids are few. Rouse and Pleijel (2001) stated that all *Sternaspis* are gonochoric with paired gonads as discrete sacs behind segment six, and that their larvae seem to be lecithotrophic and settle in less than two days, as originally reported by Child (1900) or Strathmann (1987). Consequently, the few species studied apparently lack the means to disperse long-distances because their larvae, if present, are short lived.

**Figure 1.**
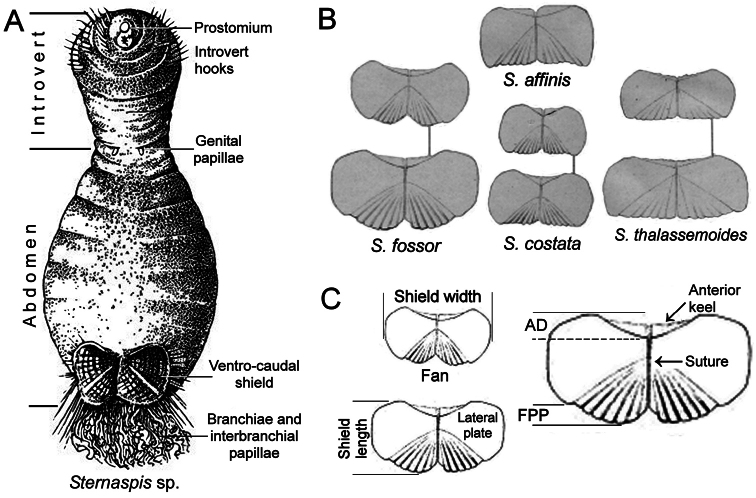
**A** *Sternaspis* sp. ventral view with some morphological features **B** Ventro-caudal shields of some *Sternaspis* species **C** Shield parts as herein proposed to distinguish different species (AD: anterior depression, FPP: fan posterior projection) (**A** modif. after Uschakov 1955, reproduced with permission; **B–C** modified after von Marenzeller 1890).

The sternaspids are capable of invaginating some anterior segments including the first three chaetigers, which often carry falcate hooks (Fig. 1A). This eversible body region is followed by another one with no chaetae in the adult stage, often carrying two fleshy ventral outgrowths, the gonopodial lobes or genital papillae, over its anterior margin. This region is followed by the often spectacular ventro-caudal shield, which has many radiating bundles of simple chaetae, often accompanied by abundant twisted filaments. Occasionally, the rectum might be prolapsed into a delicate, thin lobe.

For many polychaete groups, it has increasingly being shown that there are complications for delineating species. For sternaspids, this is a long-standing problem and even though chaetal features are diagnostic for many polychaete groups, in sternaspids they are very conservative. The first chaetigers have large, fragile hooks. The posterior region has many bundles of chaetae, but most are finely covered by thin filaments. The remaining chaetae are few in number and smooth. Consequently, the only remaining diagnostic feature is the ventro-caudal shield. The shield is usually sclerotized and can have different shapes or ornamentations. In *Sternaspis*, the shield rigidity is due to mineralized iron (Bartolomaeus 1992). Underneath the shield, there is a series of bilaterally symmetrical muscle bundles which are attached to the shield margins (Rietsch 1882), this explains why the shield is not always flat or arranged along a single plane. The chemical composition of the shield has been documented in several studies. Goodrich (1897: 240) indicated that it had no true chitin and thought their composition should resemble the same build up as chaetae. Lowenstam (1972: 157, Pl. 2) concluded that the shield includes, in decreasing abundance: FeO (33%), P_2_O_5_ (22.4%), CaO (3.4%), MgO (2.8%), BaO (0.1%) and MnO (0.04%), and that the chaetae may also contain a calcium phosphate hydrogel (Lowenstam 1972: 158). Goldberg (1974: 744) found resemblances of the iron form of sternaspids shields and the radular teeth of chitons, and regarded it as ‘mineralized by an amorphous ferric phosphate hydrogel’. Later, Lim and Hong (1996) made a study about the distribution and growth pattern in Korean *Sternaspis*. They noticed that the shield’s relative size directly depends on the body size, expressed as wet weight, but they did not study the growth pattern of the shield. This is relevant because such a study would help understand the shield’s differential expansions or variations in the ornamentation.

Because the variation of the shield’s morphological features are poorly known, its relevance as a diagnostic feature has not been widely accepted. It has been used to separate similar species (Malmgren 1867, von Marenzeller 1879, Augener 1918, Chamberlin 1919, Caullery 1944, Nonato 1966), followed with reservation (Augener 1906), or openly rejected (von Marenzeller 1890, Roule 1906, Benham 1915, Fauvel 1913, 1927, 1953, Augener 1926, Pettibone 1954, Day 1967, Fiege and Buetfering 2000), suggesting that there were few or a single cosmopolitan species. Two recent contributions have summarized the state of knowledge about sternaspids (Petersen 2000, Sendall 2006) and most of their conclusions are herein followed.

The general features of the ventro-caudal shield must be taken into account. The shield is roughly rectangular, has two lateral, symmetrical plates and is covered by a thick cuticle, especially along its margins (Vejdovský 1882: 36, Pl. 1, fig. 8). Von Marenzeller (1879) made the first fine illustration of the shield of *Sternaspis costata* and later (von Marenzeller 1890), he compared the shields in four species and their size-related variations. His illustrations are very good and useful for understanding the shield parts and their variations (Fig. 1B); von Marenzeller also gave precise localities for two species (*Sternaspis affinis* Stimpson, 1864, and *Sternaspis costata* von Marenzeller, 1879), and what he regarded as *Sternaspis scutata* (Ranzani, 1817), we are herein identifying this as *Sternaspis thalassemoides* Otto, 1821. Despite the observed differences, von Marenzeller regarded his previously described species as a junior synonym of a Mediterranean species.

The drawings of von Marenzeller (Fig. 1C) help in the understanding of variations in the general shield’s shape. The shield as a whole is usually wider than long, although individual, lateral plates tend to become wider than long in larger specimens. These lateral plates are often fused throughout their length and a suture is often visible between them, sometimes running throughout the shield, but in some other species these plates are completely fused so that sutures are not visible, or indistinct. The shield varies in different species regarding the relative shape of the anterior margins, which can be projected as rounded or acute corners, the relative curvature of the lateral margins, and especially in their posterior projection or fan. The anterior margin of each lateral shield plates has an anterior projection or keel, which is usually covered by the body wall, and this covering and the relative exposure of the anterior shield margins result in an anterior depression; this depression can be shallow as in *Sternaspis thalassemoides*, or deep as in *Sternaspis costata*. Fans are formed by the inner posterior portions of each lateral plate; the posterior margin varies in shape depending on the relative extension of the posterior corners, the relative development of the median notch, and its posterior edge. Thus, the fan’s margin can reach the shield’s posterior corners as in *Sternaspis affinis*, or *Sternaspis costata*, or markedly extend beyond them as in *Sternaspis fossor* Stimpson, 1853. The median notch can be missing as in *Sternaspis thalassemoides*, shallow as in *Sternaspis affinis*, or deep as in *Sternaspis fossor* (Fig. 1B); further, the posterior fan margin can be crenulated if the ribs marginal projections are low and round, or denticulate if these projections are sharp.

Our objectives for this study were to revise the status of all species in the genus *Sternapsis* from types or topotype specimens. This allowed us to propose emendations and redescriptions for species, and provide good illustrations. In this contribution, we first studied the morphological variation of the ventro-caudal shield in different sized specimens of a single species from the same locality and validated its usage as a diagnostic feature. On this basis, three genera are recognized and two are newly proposed. All valid species are redescribed, and three new species are recognized and described. Additionally, we include a key for all species on the basis of the form of the ventro-caudal shield plus other morphological features.

## Material and methods

Morphological variation was assessed in 30 specimens of *Sternaspis affinis* from two different localities, and the results were regarded as relevant to all other species. The stations were: 1) Southern California. RV *Velero* Station 996, Prisoners Harbor, Santa Cruz Island, 64–82 m, 12 Aug. 1939. 2) Gulf of California. Scripps Institution of Oceanography, Station P167-70, south of Isla San Pedro Martir, off San Juan (28°02.0'N, 111°47.2'W), 311–320 m, 10’ otter trawl, R. Parker, coll., 21 Mar. 1960. Four specimens from station 996 were selected for illustrations because of their size differences, but the study of variation was based on all specimens from these two stations. All specimens were carefully cleaned with a fine brush to remove fine particles on the body surface, including the shield. Specimens varied in the exposure of the eversible region and in order to standardize the measurements, only the abdominal region was measured along its maximal length (from the body constriction to the posterior body margin) and width. The shield was measured along the midventral line and the widest region of one of its lateral plates. The shield of each species was aligned in the same plane, and photographed with a digital camera. Photographs were made with different sets of cameras, microscopes and lamps, with the main objective to illustrate the diagnostic features. For some of the illustrations, a series of photos was assembled by using HeliconFocus in order to provide the best composite image as possible, but because our purpose was to illustrate diagnostic features, some non-diagnostic portions are out of focus. One specimen was subjected to standard methods for scanning electron microscopy in order to observe the fine integument papillation of the shield and it is included in the same figure. Remarks on shield growth and potential defensive or sensory roles are based upon analogies and on their relevance to gain some insight on these processes.

Type or non-type specimens of *Sternaspis* were obtained from 23 museums or institutions worldwide. The sequence for the presentation of genera is with the known genus first and then the new genera in alphabetical order. Species are presented within each genus with the type species first and then the others in alphabetic order. Because several sternaspids species have been briefly described or confused, and because no type materials were found, neotypes for eight species are proposed to objectively define the nominal taxa (ICZN 1999: 84, Art. 75), and there is an explanation for each species. In order to fulfill the qualifying criteria to clarify the taxonomic status for the nominal taxa, we provide keys to identify genera, and all species per genus, and describe and illustrate neotype specimens, redefine the species morphological features to emphasize the distinction from similar species in the key, and certify that all specimens are deposited in recognized scientific institutions. When more specimens were available, some were regarded as ‘paraneotypes’; although not recognized by the ICZN it is a useful concept for recognizing specimens collected from the same general location as the neotypes (e.g. Evenhuis 2008, Hawksworth 2010).

AMAustralian Museum, Sydney, Australia.


ANSPAcademy of Natural Sciences of Philadelphia, Philadelphia, USA.


CASCalifornia Academy of Sciences, San Francisco, USA.


CMNHCoastal Branch of Natural History Museum and Institute, Chiba, Japan.


ECOSURColección de Referencia, El Colegio de la Frontera Sur, Chetumal, México.


HMCSHuntsman Marine Science Centre, Atlantic Reference Centre, St. Andrews, Canada.


IMNHIcelandic Institute and Museum of Natural History, Reykjavik, Iceland.


IRFAInstitut de Recherche Foundamentale et Appliquée, Université Catholique de l’Ouest, Angers, France.


LACMNatural History Museum of Los Angeles County, Allan Hancock Polychaete Collection, Los Angeles, USA.


MCEMMuseu do Centro de Estudos do Mar, Laboratory of Benthic Ecology, Parana, Brazil.


MNHLNaturalis Biodiversity Cener (formerly National Museum of Natural History), Leiden, The Netherlands.


MNHNMuseum National d’Histoire Naturelle, Paris, France.


NHMThe Natural History Museum, London, England.


NHMWNaturhistorisches Museum Wien, Austria.


PMBCPhuket Marine Biological Center, Phuket, Thailand.


RBCMRoyal British Columbia Museum, Victoria, Canada.


SIOScripps Institution of Oceanography, La Jolla, USA.


SMNHSwedish Museum of Natural History, Stockholm, Sweden.


UMMLMuseum of Marine Invertebrates, University of Miami, Rosenstiel School of Marine and Atmospheric Science, Miami, USA.


UNAMColección de Referencia de Invertebrados Bentónicos, Unidad Académica Mazatlán, UNAM, Mazatlán, México.


USNMSmithsonian Institution, National Museum of Natural History, Washington, USA.


ZIRASZoological Institute, Russian Academy of Sciences, Sankt-Peterburg, Russia.


ZMAPolychaete Collection, Zoological Museum at the University of Amsterdam (transferred to Naturalis Biodiversity Cener, Leiden), The Netherlands.


ZMUCZoologisk Museum, University of Copenhagen, Denmark.


## Results

### Morphological characters

Sternaspids are segmented and many segments carry chaetae, but counting segments has been difficult because the anterior region is eversible; if exposed it can be variously contracted, and several segments lack chaetae in the adult stage. Traditionally, segment counting included the prostomium and peristomium (Vejdovský 1882: 36), which is incorrect, and there are discrepancies regarding the peristomial extent, such that what has been regarded as segment 3 should be segment 1, discounting prostomium and peristomium. The peristomium has been regarded as restricted to the area surrounding the mouth (Hutchings 2000: 224), or a whole segment surrounding the prostomium (Goodrich 1897: Pl. 16,fig. 16; Rouse and Pleijel 2001: 229); the latter concept has been followed here. It must be also taken into account that chaetal bundles are displaced posteriorly on the first three chaetigers (Rietsch 1882: 6). However, the traditional counting has been followed to facilitate comparisons with previous publications.

The body of sternaspids has three main regions. The introvert includes the head and the thorax; it is eversible, extending from the prostomium and peristomium to segments 5–6. Its integument is thin, delicate, and the first three chaetigers carry falcate hooks. The constricted region often includes only segments 7–8, carries the genital papillae, and is followed by an expanded abdomen, which is the largest part of the body. The anterior abdomen has some segments without chaetae in the adult stage, whereas the posterior abdomen carries the ventro-caudal shield, its associated marginal chaetae, and two dorsal groups of branchial filaments, usually arranged in discrete branchial plates.

There are three body patterns among the *Sternaspis* species concerning their shield type, introvert hooks, and the number of abdominal segments. The first pattern includes most species. They have shields with a continuous, stiff layer often carrying radial ribs, concentric lines, or both, and their sediment cover is easily brushed off; their introvert hooks are cylindrical, tapered, and their anterior or pre-shield abdomen has seven segments. This pattern is regarded as *Sternaspis* *sensu stricto*.

The second pattern includes two species, one being newly described below, and their shields are remarkably soft, without ribs or concentric lines, but their sediment cover is firmly adhered such that it cannot be brushed off; their introvert hooks are cylindrical, tapered, and their anterior or pre-shield abdomen has seven segments. For this pattern, *Caulleryaspis* gen. n. is being proposed.

The third pattern also includes two species with one newly described below. Their shields are stiff, with ribs but without concentric lines, and their sediment cover is easily brushed off; their introvert hooks are subdistally expanded, or spatulate, and their anterior or pre-shield abdomen has eight segments. *Petersenaspis* gen. n. is being proposed to include these two species. The relevant morphological features in sternaspids are shown in Table 1.

**Papillae**. There are five basic types of papillae along the body, but because they are delicate and easily eroded, their apparent abundance could depend on their density and/or the general sample treatment. The papillae can be separated into body papillae, mouth papillae, genital papillae, shield papillae and interbranchial papillae. Body papillae are present over at least part of the body surface on all species and may have different arrangements. They may be evenly distributed over most of the body, either very densely as in *Sternaspis fossor*, sparsely distributed as in *Sternaspis scutata*, restricted to a particular body region or area, or in one or two transverse rows of clusters or ‘pompoms’ on some or most segments, as for *Sternaspis africana* Augener, 1918 n. status and *Sternaspis costata*. Mouth papillae surround the mouth opening and are usually more resistant than other papillae present nearby. The largest papillae are the genital papillae; they are a pair of large, muscular, often extended conical papillae protruding from the septum between segments 7–8. Where they were not apparent, either because they have been lost or are invaginated, the pores through which they extend out could usually be detected. For some species such as *Sternaspis fossor* and *Sternaspis affinis* these papillae are short and narrow, whereas in other species such as *Sternaspis scutata*, they are much longer, extended and broader. The ventro-caudal shield is covered by integument and it has many papillae protruding from the surface. On some species they can be short or filamentous, recurved or projecting at right angles from the shield. On larger individuals these are often worn off or missing through abrasion; however, the presence of fine sediment particles provides an indirect indication of their abundance. The interbranchial papillae are long, white filaments that occur on the cuticle of the caudal end among the branchiae of most species. They are distinctive from the branchiae being more slender and with the appearance of white hair, whereas the branchiae are much thicker, regularly coiled or curved and tan or blond in preserved organisms.

**Ventro-caudal shield**. The shield is bilaterally symmetrical, wider than long. The three main features to be taken into account are the apparent texture or appearance, stiffness, and the variations in the relative development of shield regions. It must be emphasized that shields are not fully tangential to the body and that their different parts are not aligned along a single plane; the anterior margins and the fan are more or less along the same plane but the lateral margins are often depressed and this can be combined with a variable intensity of body contraction, such that they can distort the shield’s perspective. This is especially true of the lateral margins, which when strongly contracted appear straight although they are rounded; consequently, it is important that the specimens and their shields should be arranged such that the shield’s surface is as horizontal as possible, trying to set the anterior and fan margins along the same plane. If specimens are strongly contracted and lateral shield plates are pulled dorsally, then one of the plates should be chosen to be observed and photographed.

Texture and appearance. The shield surface can carry variable amounts of sediment particles; these particles must be carefully brushed off to reveal the surface. Once the sediment has been removed, the surface can be almost completely smooth, with diagonal radial ribs, concentric lines, or both. These features can be poorly developed or difficult to see, and these differences are used here to separate similar species. Concentric lines are usually visible, but may be faint or similar in colour to the remainder of the shield; although these are most likely added as the body and shield size increase, it is unknown if they are added at regular intervals and if they can be used to age individuals. There are size-related morphological trends within the same species. The shields of young individuals are rounder and flatter compared to those of larger adults of the same species. The ribs are associated with the bundles of chaetae protruding from under the shield; as body size increases, the prominence of the ribs also increases. The colour of the shield is quite variable and inconsistent ranging from a sulfur yellow to rust-red, and even green to purple-black. The concentric lines also vary in colour and may even appear to cause a discontinuity in the colour of the shield; however, their presence is consistent within a species.

Stiffness. As indicated above, the shield is generally rigid and brittle among *Sternaspis* and *Petersenapis* species, whereas in *Caulleryaspis* it is soft and easily bent.

**Chaetae**. There are five basic types of chaetae. All species of *Sternaspis* and *Caulleryaspis* have pointed, tapered introvert hooks, whereas in *Petersenaspis* they are subdistally expanded. The number of chaetae in each bundle increases with body size and age. It is unclear if these chaetae originate as neurochaetae, notochaetae or a combination of both. Vejdovský (1881) considered them as both noto- and neurochaetae. Petersen (2000: 315) noticed what she thought were very small notochaetae close to the dorsalmost hooks. Because more detailed studies are needed to clarify this issue, including examining early juveniles and ontogenetic studies, no distinction is made or proposed here. The pre-shield capillary chaetae can usually be found as few (1–2) short, delicate simple chaetae along the dorsolateral surface of segments 8–15. On some individuals, especially larger ones, these may not protrude from the epidermis or may have been broken or worn off. If the corresponding area is viewed carefully from above segments 8–15 using a dissecting microscope, they can usually be found. Although no evidence or proposal to date has been made to suggest that these are notochaetae or neurochaetae, their dorsolateral position suggests they are notochaetae. The most prominent chaetae are the ventro-caudal shield chaetae; they protrude from the underside of the ventro-caudal shield in fascicles of chaetae consisting of three types: 1) Stout, hirsute capillaries on which sediment particles strongly adhere; most of the bundles of chaetae consist of this type and comprise the counts of lateral and posterior fascicles; 2) Very long slender, smooth, capillary chaetae found as couplets or triplets included in the most posterolateral fascicle which once broken are regarded as peg chaetae; and 3) Adjacent and medial to the peg chaetae, a small group of short, delicate smooth capillaries is also present.

The bundles of shield chaetae are divided into 9–11 lateral and usually 5–7 posterior fascicles but they are fragile; in one species, only 3 posterior bundles were observed. The lateral bundles consist of longer chaetae with each consecutive bundle longer than the previous one as progressing from the anteriormost to the posteriormost bundle. The last few lateral bundles can be very close together, and can even appear to overlap. Unless the groups are viewed laterally to detect the point of insertion, two or more groups can be misinterpreted as being only one. The posterior bundles are more similar to each other in length than the lateral bundles. At the point on the cuticle where each lateral or posterior fascicle emerges, the individual chaetae within a fascicle can form one of four arrangements: 1) oval or circular; 2) a curved line with each fascicle in line with the next; 3) an offset line with each fascicle parallel to the next; or 4) an offset straight line with each fascicle parallel to the next.

Peg chaetae. These are apparently fused or congealed short chaetae on the ventro-caudal shield posterior corners, between the most lateral posterior chaetal bundle and the most posterior lateral chaetal bundle. Intermixed with the congealed chaetae may be a few much longer capillaries dorsal to the peg chaetae themselves. Although Sluiter’s (1882) description of *Sternaspis spinosa* included the first mention of the ‘peg chaetae’ and was one of the main characters forming the basis of his description, they have been observed on all species with the exception of *Sternaspis capillata* (Nonato, 1966) comb. n. Although on some individuals it appeared that one or both of the peg chaetae were missing, or had been broken off. The form of the peg chaetae varies at least within populations. On some larger individuals peg chaetae are comparatively more robust and stout at the base where the chaetae emerge from the cuticle. The oblique, often larger rib radiating from the center of the shield is associated with the peg chaetae, which are placed under the ventro-caudal shield along the same path as the rib. This probably accounts for von Marenzeller’s (1879) description of the shield for *Sternaspis costata* as having more than two parts. The length of the chaetae that comprise the peg chaetae and the collective width of those chaetae at the base are the two main differences observed between individuals. The colour of these chaetae varies from golden to bronze. Some species have filamentous papillae associated with the branchiae, or abundant sediment attached to the ventro-caudal shield area, both of which can make the peg chaetae difficult to locate. Further, adjacent and medial to the peg chaetae, if present, there is a small group of delicate, short, smooth capillary chaetae. Similar to the situation with the peg chaetae, these can be difficult to locate when hidden by filamentous papillae or adhered sediment in the area of the ventro-caudal shield.

**Branchial plate**. Branchial filaments and interbranchial papillae are arranged into two groups placed on each side of the anus. The filaments are usually densely packed and arranged on well defined branchial plates, which are basally expanded, becoming more acute towards the distal portion; the plates may even be darker than the surrounding integument. However, in only one known species (*Petersenaspis capillata* (Nonato, 1966) comb. n.), branchial bases are not so densely packed, interbranchial papillae are less abundant, so branchial plates are not well defined, and the integument has the same colour as adjacent regions.

**Table 1.** Main morphological features of *Sternaspis* Otto, 1821, *Caulleryaspis* gen. n. and *Petersenaspis* gen. n. species [shield features are surface (S), anterior margins (A), anterior depression (D), lateral margins (L), and fan (F)].

**Species**	**Bodypapillae**	**Shield**	**Lateral shield chaetae**	**Posterior shield chaetae**	**Peg chaetae**
*Sternaspis affinis*	Rows of clusters	S ribs and conc. lines. A rounded. D deep. L rounded. F crenulated.	10 in oval pattern	5 in linear pattern	Present
*Sternaspis africana* stat. n.	Rows of clusters	S ribs. A angular. D deep. L rounded. F denticulated, crenulated in larger specimens.	9 in oval pattern	5 in slightly curved pattern	Present
*Sternaspis andamanensis* sp. n.	Absent	S barely ribs. A angular. D deep. L rounded. F denticulated, notched laterally.	9 in oval pattern	5 in linear pattern	Present
*Sternaspis costata*	Two rows of clusters	S ribs and conc. lines. A rounded. D shallow. L rounded. F deeply notched.	10 in oval pattern	5 in roughly linear pattern	Present
*Sternaspis fossor*	Abundant, evenly distributed, rows of clusters	S ribs, tenuous conc. lines. A rounded. D deep. L rounded. F notched, smooth.	10 in oval pattern	7 in linear arrangement	Present
*Sternaspis islandica*	Rows of clusters	S ribs and conc. lines. A rounded. D deep. L rounded. F truncate, notched, smooth.	10 in oval pattern	6 in oval pattern	Present
*Sternaspis maior*	Abundant, evenly distributed	S ribs, no conc. lines. A rounded. D shallow. L rounded. F crenulated.	10 in oval pattern	7 in linear pattern	Present
*Sternaspis princeps*	Evenly distributed	S ribs, no conc. lines. A rounded. D deep. L rounded. F truncate, crenulated.	10, pattern unknown	6, pattern unknown	Present
*Sternaspis rietschi*	Probably evenly distributed	S covered. A rounded. D shallow. L rounded, crenulated. F truncate, crenulated.	10, pattern unknown	5, pattern unknown	Present
*Sternaspis scutata*	Evenly distributed	S ribs and conc. lines. A truncate. D deep. L straight. F projected, smooth.	10 in oval pattern	6 in an arc	Present
*Sternaspis spinosa*	Rows of clusters	S barely ribbed, conc. lines. A angular. D shallow. L rounded. F truncate, crenulated.	10 in curved pattern	5, in oval pattern	Present
*Sternaspis thalassemoides*	Probably evenly distributed	S ribs, conc. lines. A rounded. D deep. L rounded. F truncate, crenulated.	10 in oval pattern	6, in oval pattern	Probably present
*Sternaspis thorsoni* sp. n.	Abundant, evenly distributed	S ribs and conc. lines. A rounded. D deep. L rounded. F crenulated.	10 in oval pattern	7, in oval pattern	Present
*Caulleryaspis gudmundssoni* sp. n.	Evenly distributed	S with sediment particles adhered. A rounded. D deep. L rounded. F truncate.	10 in linear pattern	3 in oval pattern	Present, apparently emerging from the shield
*Caulleryaspis laevis* comb. n.	Evenly distributed	S with sediment particles adhered. A rounded. D shallow. L rounded. F truncate, smooth.	10 in oval pattern	5 in offset linear pattern	Present
*Petersenaspis capillata* comb. n.	Evenly distributed	S ribs barely visible. A rounded. D shallow. L rounded. F without lateral notches.	11 in oval pattern	10 in oval pattern	Absent
*Petersenaspis palpallatoci* sp. n.	Evenly distributed	S ribs distinct. A acute. D deep. L rounded. Fwith lateral notches.	11 in oval pattern	10 in oval pattern	Absent

### Intra-specific variation in *Sternaspis affinis*

**Figure 2.**
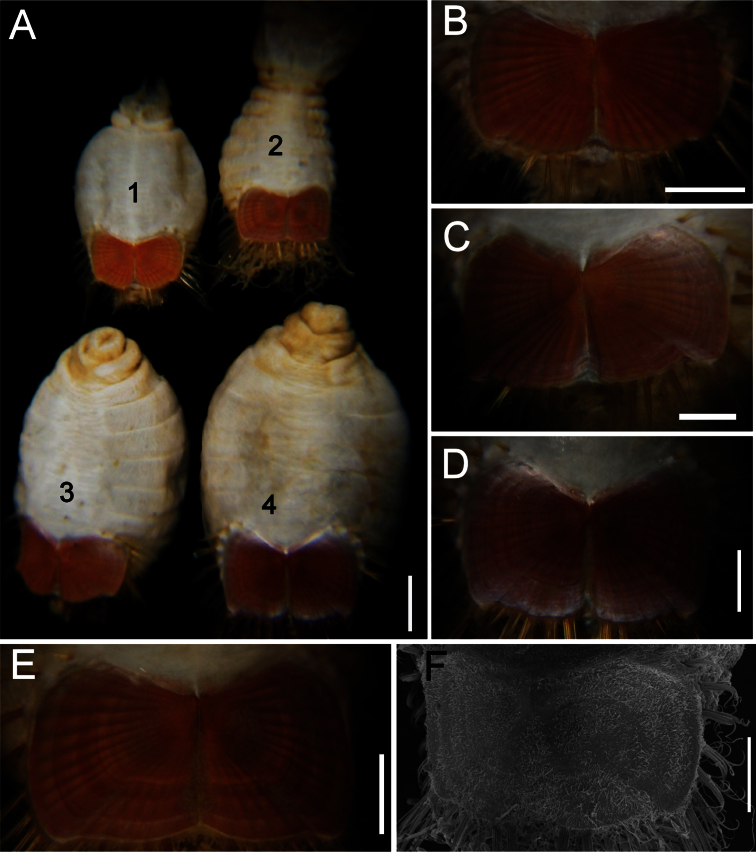
Variation of the ventro-caudal shield in *Sternaspis affinis* Stimpson, 1864, station 996 **A** Four specimens showing size differences **B** Specimen 1, ventro-caudal shield **C** Specimen 3, ventro-caudal shield **D** Specimen 4, ventro-caudal shield **E** Specimen 2, ventro-caudal shield **F** Same, ventro-caudal shield showing integument papillae. Bars: **A** 2 mm, **B–F** 1 mm.

The 30 specimens identified as *Sternaspis affinis* exhibited the following variations. The shield size depends on body size and each plate is usually wider than long (Fig. 2A). The shield is rectangular, has well-developed radial ribs and concentric lines, and it is completely covered by a thick cuticle provided with abundant, thin papillae (Fig. 2F), such that the shield’s ornamentation is not actually exposed, but it is visible because of the cuticle transparency. The anterior margins are angular and the anterior keels are not exposed; the lateral margins are slightly expanded medially, curved, whereas the fan is slightly expanded beyond the posterior corners, being smooth in smaller specimens (Fig. 2B, E), becoming crenulated in larger specimens and with a lateral notch (Fig. 2C, D). Each lateral plate has a large, diagonal ridge or rib forming the posterior corners.

**Figure 3.**
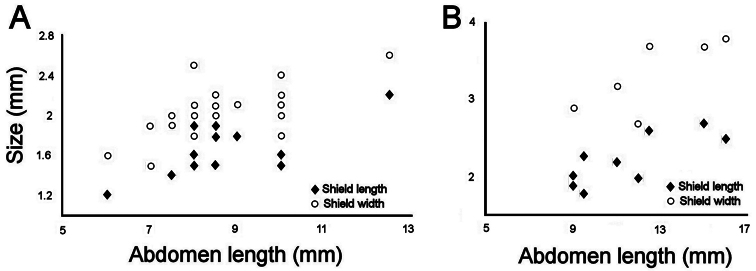
Relationship between body size (abdomen length) and ventro-caudal shield size (left plate) **A** Southern California lot (LACM 3025) **B** Gulf of California lot (LACM 3026).

Station 996 (LACM 3025). There were 24 grayish specimens, but only four (17%) had their anterior end exposed. These few specimens have 10–13 falcate, golden hooks per bundle with darker subdistal areas and they increase in size and number with increasing body size. Their abdomen was 6.0–12.5 mm long, 4–8 mm wide, whereas the shield was 1.2–2.2 mm long, 1.5–2.6 mm wide (Fig. 3A). The pigmentation was pale brown to pale orange, often with paler concentric bands. The posterior margin is smooth in small specimens and becomes more crenulated with increasing body size. The shield had 7–10 lateral and 6–7 posterior fascicles of golden chaetae, but the shield posterolateral corners have two fascicles, one above the other, being the last lateral and the first posterior ones. In some specimens, what has been regarded as ‘peg chaetae’ were observed but they are actually the broken bases of very delicate, thin capillary chaetae that can be present also in the adjacent posterior chaetal bundles. The body papillae were mostly eroded, with few specimens showing long abundant papillae, but most had papillae restricted to some transverse groups, especially visible along the dorsal surface of posterior segments.

Station P-167-70 (LACM 3026). There were eight larger yellowish specimens, four had the anterior end exposed, two had it partially exposed and the other two did not expose it at all. The specimens with exposed anterior end were 14–23 mm long and 7–12 mm wide; they had 10–15 falcate, bronze neurochaetae with darker subdistal areas in the second chaetiger, and their number and size depends on body size. Their abdomen was 9–16 mm long and 6–12 mm wide, whereas the shield was 1.8–2.7 mm long and 2.3–3.8 mm wide (Fig. 3B). The pigmentation pattern was pale brown or reddish, often with paler concentric bands. The fan was smooth in smaller specimens becoming barely crenulated in medium-sized ones, and crenulated in the three larger specimens. The shield had 9–10 lateral fascicles and 7 posterior fascicles of bronze chaetae. ‘Peg chaetae’ were noticed in about half the specimens, often some delicate, thin, very long chaetae were still stemming from the chaetal lobe. The body papillae were visible as eroded groups, especially along the posterior dorsal surface.

### Further comments

**Taxonomic features**. Body papillae appear to be abundant throughout the body but they are very delicate and usually only the remains of some transverse groups can be noticed along the dorsal, posterior surface. They are usually covered with fine sediment particles in *Sternaspis affinis* but in other species these papillae might adhere larger particles and this feature may be useful to separate similar species.

The falcate hooks in anterior chaetigers are more abundant in larger specimens but often their anterior end is not exposed, which reduces their usefulness as a diagnostic feature. The inner organization of the subdistal region in larger chaetae might be useful as the septa apparently take on different shapes, but this has not been further evaluated because the tips of these chaetae are not always exposed.

The ventro-caudal shield has an external integument layer with abundant papillae which retain some fine sediment particles. The relative proportion of the lateral plates and their subplates (lateral and posterior), together with their ornamentation is rather consistent and independent of body size, as originally (and indirectly) indicated by von Marenzeller (1890); however, the fan margin varies from a smooth surface to barely crenulated in medium-sized specimens, becoming crenulated in larger specimens. By transparency, the shield shows ribs and concentric growth lines, although the pigmentation pattern might be solid or with some concentric paler bands. Consequently, the relative transparency of the shield integument, together with the shield shape, relative development of the margins, and the surface ornamentation are consistent and should be used as diagnostic features. They are used in the keys below.

Fauchald and Rouse (1997) reported that the chaetae associated with the shield consist of notochaetae only. Each bundle appears to originate from one of a series of closely associated source points under the ventro-caudal shield, very close to the median of the body. We were unable to discern if the bundles consisted of notochaetae, neurochaetae or both. However, each bundle is associated with a separate segment.

The peg chaetae are the broken bases of long, delicate capillary chaetae in the first (and second) posterior fascicle(s). As stated above, they have been noticed before and even called spines; however, they were regarded as the remains of long chaetae by Petersen (2000: 316) and we concur with this. They are very fragile and can be completely eroded giving the impression that these long, delicate capillaries are not present at all. Consequently, their taxonomic relevance must not be over-emphasized but we include them below in order to provide standardized descriptions.

**Growth patterns**. There are few examples of continuous growth in bilaterally symmetrical body parts arranged along a single plane, such as is the case for *Sternaspis* shields. One of the best detailed studies involves dicots leaf growth. It has been found that in complex structures, the growth is mostly differential, with some regions growing continually whereas other regions have an arrested development, and these variations are regulated by a series of growth genes, including some others for vein patterns (Tsukaya 2002). The overall result shows some variations in leaf shape or venation, but these variations are within a single pattern, and leaves often are reliable for separating closely related plant genera or species. Sternaspid shields show differential growth as well and the posterior region is the most variable because it may be smooth or crenulated, but its relative shape and size-relation to the lateral lobes is of a consistent pattern. Consequently, we suggest that its general shape is useful to separate species within genera.

Flat or depressed shells showing variations in shape and ornamentation are frequent among bivalves, especially among the superfamily Pectinoidea. The presence of lateral projections and the relative development of ribs and growth lines are commonly employed to separate families, genera and species, and even the height: length proportion has taxonomic relevance (Coan et al. 2000). We suggest that a similar approach can be used in the taxonomy of sternaspid polychaetes, and we use the shield features to separate species in the key below.

**Defensive or sensory roles**. Phragmosis is ‘a method of closing the burrow or nest by means o some specially adapted part of the body (as the flattened head in some ants)’. The word combines the Greek words *phragmos* or fence, and the new Latin *osis*. This role has been noticed in several different animal groups and the name appears in many taxa. The term was introduced by Wheeler (1927) on the basis of several examples of ant genera and some spiders and frogs (Toledo et al. 2011). Phragmosis is widespread among tube-dwelling polychaetes and the closing device is an operculum, like in sabellariids or serpulids, or into cephalic or anal plates like in maldanids. In the latter, there might be a series of cirri which might surround the anus; this modification has been also noticed in some opheliids or capitellids, and its sensory role has been documented (Purschke 2005). In sternaspids, the sensory role may rely upon the marginal shield appendages themselves, such as the interbranchial papillae and the long, delicate, thin chaetae that are sometimes visible in some specimens. A similar pattern of marginal sensory chaetae has been documented in *Cyclocosmia* Ausserer, 1871, a ctenizid spider with a highly modified abdomen (Zhu et al. 2006).

The sternaspid ventro-caudal shield is not exposed and as such may have no direct defensive role, or only a slight one if any at all. Rather it may function as an anchoring device for the muscular bundles in the posterior body region. However, combined with the marginal chaetal bundles, they may form a defensive structure to protect the ingestion of the posterior body region. The presence of long spines as an anti-predatory modification has been widely documented in the fossil record, among several benthic groups, including infaunal taxa (Brett and Walker 2002, Walker and Brett 2002).

A study by Wheeler (1927) helps understand the relationship between muscle bundles and the shape of the ventro-caudal shield; he concluded that the ‘form of the (ant’s) head and face is very largely determined by the size and shape of the flexor muscles of the mandibles and in turn the functional or adaptative peculiarities of these organs are closely correlated with the character of the flexor muscles.’ He also showed that the head and face of a single species varies depending on the relative size of the corresponding castes, but with the exception of the sexually active members, which are usually wasp-like, the head and face of all castes of the same species varies within a certain pattern (Wheeler 1927, figs 10, 11). We believe that a similar pattern of muscular development must operate to modify the appearance of the ventro-caudal shield in sternapids, and that the variations found would fall within a consistent range such that it can be used to discriminate specimens into different species.

## Systematics

### Order Sternaspida Dales, 1962

#### Family Sternaspidae

Carus, 1863

http://species-id.net/wiki/Sternaspidae

##### Type genus.

*Sternaspis* Otto, 1821.

##### Diagnosis.

Body peanut-shaped. Introvert with falcate, tapered or subdistally expanded hooks. Segments 7–8 constricted, with genital papillae protruding ventrally. Pre-shield region with 7 or 8 segments. Ventro-caudal shield usually stiff, often provided with radiating ribs and concentric lines, rarely flexible. Marginal shield chaetal fascicles include lateral and posterior chaetae, sometimes peg chaetae or additional delicate chaetae present. Branchiae coiled, abundant filaments, emerging from two lateral dorsal plates, near the anus, or directly from the body wall. Additional, thinner coiled interbranchial papillae present.

**Composition**. Three genera: *Sternaspis*, *Caulleryaspis* gen. n. and *Petersenaspis* gen. n.

##### Remarks.

Vejdovský (1882) published a very thorough account of the anatomy, physiology and development of *Sternaspis scutata*; only a few months later, Rietsch (1882) published an equally thorough account of the same species. The reason *Sternaspis* was given so much attention was likely due to the argument over the distinction between “Gephyrea” and Chaetopoda within Annelida, and that *Sternaspis* had attributes that pertained to both groups. In general, *Sternaspis* does resemble an echiurid from the exterior, even more so if one confuses the anterior end with the posterior, as was the case until corrected by Krohn (1842). Vejdovský (1882) and Rietsch (1882) outlined the affinities aligning *Sternaspis* with the polychaetes and shortly afterwards *Sternaspis* was accepted as a polychaete (Dahl 1955).

The family was proposed by Carus (1863: 453) and one hundred years later, it was regarded as forming an independent order by Dales (1962). This proposal was accepted by Fauchald (1977), Pettibone (1982), George and Hartmann-Schröder (1985), and Hartmann-Schröder (1996). An analysis of morphology and six genes (Zrzavý et al. 2009) did not clarify the affinities for sternaspids because different approaches gave different topologies or affinities. Thus, their Bayesian combination indicates Sternaspidae are a sister group to a clade including sabellids-serpulids, sabellariids, and *Trochochaeta*-Spionidae-*Poecilochaetus*. The unweighted maximum-parsimony indicates they form a clade with sabellariids, which is a sister group to Sabellidae and *Trochochaeta*-Spionidae-*Poecilochaetus*. The weighted maximum-parsimony indicates they group with Fauveliopsidae, and together become a sister group for Sabellidae- Serpulidae, which is a sister group to Sabellariidae and the other grouped taxa of former analysis.

##### Key to genera of Sternaspidae Carus, 1863

**Table:** 

1	Ventro-caudal shield stiff	2
–	Ventro-caudal shield soft, covered by adhered sediment particles; without ornamentation; introvert hooks tapered; anterior abdomen with 7 segments	*Caulleryaspis* gen. n.
2(1)	Introvert hooks tapered; shield ornamentation includes ribs, concentric lines, or both; anterior abdomen with 7 segments	*Sternaspis* Otto, 1821, restricted
–	Introvert hooks subdistally expanded or spatulate; shield ornamentation with ribs but no concentric lines; abdomen with 8 segments	*Petersenaspis* gen. n.

#### Genus Sternaspis

Otto, 1821, restricted

http://species-id.net/wiki/Sternaspis

##### Type species.

*Sternaspis thalassemoides* Otto, 1821, by monotypy.

##### Diagnosis.

Sternaspids with introvert hooks falcate, tapered. Pre-shield region with 7 segments. Ventro-caudal shield stiff, usually with radial ribs and concentric lines. Branchial filaments arranged in discrete branchial plates.

##### Remarks.

A species resembling current *Sternaspis* was described and illustrated by Janus Plancus in 1760 as a sea cucumber under the name *Mentula Cucurbitacea Marina* in a book on Mediterranean marine animals (Plancus 1760). In that account Plancus indicated that the specimen was from near Rimini, the Emilia-Romagna Italian region bordering the Adriatic Sea. From the description and accompanying illustration, he was undoubtedly describing a sternaspid. Plancus apparently neglected to use binomial nomenclature in his work and so his name is not available (Petersen 2000).

The next described species in the group was *Echinorhynchus scutatus* Renier (1807). Petersen (2000) indicated that Renier’s paper, or what could be found of it, was rejected as a formal publication by the International Commission of Zoological Nomenclature (ICZN 1954), although some names have been officialy validated (Muir and Petersen 2010). Ten years after the account by Renier, the first valid description of a species was published by Ranzani (1817) as *Thalassema scutatus*.

Otto (1821) proposed *Sternaspis* to replace *Thalassema* Ranzani, 1817, and described *Sternaspis thalassemoides*, which he regarded as closely allied to *Thalassema scutatus*. Otto indicated that *Thalassema* had been already employed by Pallas (and replaced by Leach 1816, to *Thalessema*). The type species for *Sternaspis* has been regarded as *Echinorhynchus scutatus* Renier, 1807 by Hartman (1959), Fauchald (1977) and Gilbert (1984). This is incorrect because of the rejection of the publications by Renier, and because the only species included in the proposal of the new genus was *Sternaspis thalassemoides* Otto, 1821. Consequently, this must be regarded as the type species by monotypy. Although Ranzani had understood correctly the body ends, Otto confused them because he thought the shield was anterior. Claparède (1869) praised Krohn (1842) and Müller (Mueller 1852) for setting it straight as to which end of sternaspids was anterior and which posterior. However, it seems that the first indication of the correct body polarity was made by de Blainville (1828: 500–501, Pl. 26, unnumb.), because he corrected the illustrations, although he repeated the confusions regarding the body features.

*Sternaspis* differs from *Petersenaspis* gen. n. because the ventro-caudal shield is stiff, the introvert hooks are tapered, not subdistally expanded, and the branchial filaments are arranged in discrete plates, not loosely arranged. *Sternaspis* differs from *Caulleryaspis* gen. n. because the latter has a soft ventro-caudal shield with abundant sediment particles on it.

*Sternaspis* includes, besides the type species, *Sternaspis thalassemoides* Otto, 1821 reinstated, from the Mediterranean Sea, *Sternaspis affinis* Stimpson, 1864 from the Northeastern Pacific, *Sternaspis africana* Augener, 1918, new status, from Western Africa, *Sternaspis andamanensis* sp. n. from the Andaman Sea, *Sternaspis costata* Marenzeller, 1879 from Japan, *Sternaspis fossor* Stimpson, 1853 from the Northwestern Atlantic, *Sternaspis islandica* Malmgren, 1867 from Iceland, *Sternaspis maior* Chamberlin, 1919 from the Gulf of California, *Sternaspis princeps* Selenka, 1885 from New Zealand, *Sternaspis rietschi* Caullery, 1944 from abyssal depths around Indonesia, *Sternaspis scutata* (Ranzani, 1817) from the Mediterranean Sea, *Sternaspis spinosa* Sluiter, 1882 from Indonesia, and *Sternaspis thorsoni* sp. n. from the Arabian Gulf. In *Petersenaspis* gen. n., besides the type species, *Petersenaspis capillata* (Nonato, 1966) comb. n. from Central and Southern Brazil, the genus also includes *Petersenaspis palpallatoci* sp. n. from the Philippine Islands. *Caulleryaspis* gen. n. includes *Caulleryaspis gudmundssoni* sp. n. from Iceland and *Caulleryaspis laevis* (Caullery, 1944) comb. n. from Indonesia.

##### Key to species of *Sternaspis* Otto, 1821

(distribution in parenthesis after studied materials)

**Table:** 

1	Ventro-caudal shield’s fan with a distinct median notch	2
–	Ventro-caudal shield’s fan continuous, without a distinct median notch	6
2(1)	Shield with radial ribs and concentric lines distinct	3
–	Shield with radial ribs distinct, concentric lines barely visible	*Sternaspis maior* Chamberlin, 1919 (Eastern Pacific, Gulf of California)
3(2)	Fan with median notch shallow	4
–	Fan with median notch deep; shields usually with concentric bands	5
4(3)	Shield with distinct concentric bands; main rib and posterior corners directed posteriorly	*Sternaspis affinis* Stimpson, 1864 (Northeastern Pacific Ocean)
–	Shield without concentric bands; posterior corners directed laterally	*Sternaspis scutata* (Ranzani, 1817) (Mediterranean Sea and Northeastern Atlantic Ocean)
5(3)	Shield with posterior corners distinct	*Sternaspis costata* von Marenzeller, 1879 (Northwestern Pacific Ocean)
–	Shield with posterior corners poorly-defined	*Sternaspis fossor* Stimpson, 1853 (Northwestern Atlantic Ocean)
6(1)	Fan margin crenulated, not projected posteriorly	7
–	Fan margin denticulated, medially projected posteriorly	11
7(6)	Shield with ribs and concentric lines	8
–	Shield with ribs; concentric lines indistinct	*Sternaspis princeps* Selenka, 1885 (Southwestern Pacific Ocean, New Zealand)
8(7)	Shield anterior margins rounded	9
–	Shield anterior margins acute	*Sternaspis spinosa* Sluiter, 1882 (Indonesia, Java)
9(8)	Shield with posterior corners distinct	10
–	Shield with posterior corners indistinct	*Sternaspis rietschi* Caullery, 1944 (Indonesia)
10(9)	Posterior corners barely projected beyond fan margin; introvert hooks thick, bronze	*Sternaspis thalassemoides* Otto, 1821 (Northeastern Atlantic Ocean and Mediterranean Sea)
–	Posterior corners projected beyond fan margin; introvert hooks thin, golden	*Sternaspis thorsoni* sp. n. (Indian Ocean, Arabian Gulf).
11(6)	Fan without lateral notches; body papillae arranged in distinct transverse rows	*Sternaspis africana* Augener, 1918 n. status (Eastern Atlantic Ocean, Ghana to Angola)
–	Fan with lateral notches; body papillae distributed homogeneously, not arranged in transverse rows	*Sternaspis andamanensis* sp. n. (Indian Ocean, Andaman Sea)

#### Sternaspis thalassemoides

Otto, 1821, reinstated

http://species-id.net/wiki/Sternaspis_thalassemoides

Figures 1B 4

Sternaspis thalassemoides Otto, 1821: 619–627, Pl. 50, figs 1–5; delle Chiaje 1822:Pl. 62, fig. 18 (upside down; no details of shield or chaetae), 1831: 204 (legend for plate 62, figure 18), 1841(3): 76–79, Pl. 43 (legend for plate 62, figure 4), Pl. 94 (for plate 84), Pl. 106 (for plate 62, figure 18); Krohn 1842: 426-432; de Quatrefages 1866: 590–591; Goodrich 1897: 233–245, Pls. 15–16, figs 1–24.
Sternaspis scutata : Vejdovský 1882: 33–90, Pls. 1–10; von Marenzeller 1890: 5–8, Pl. 1, fig. 6 (*non*Ranzani 1817).
Sternaspis assimilis Malmgren, 1867: 195–196.

##### Type material.

**Italy**, **Tyrrhenian Sea, Naples**. Neotype (ZMUC POL-2159) and 3 paraneotypes (ZMUC POL-2160), 1928, no further data.

##### Additional material.

**Italy. Tyrrhenian Sea, Bay of Naples**. 3 spec. (ANSP 1880). 1 spec. (SMNH 50759). **Adriatic Sea**. 2 spec. (ECOSUR 2642), Sta. 167 (no coord.), 1-VIII-1966. 1 spec. (ECOSUR 2644), Sta. 151 (no coord.), 1966.

##### Description.

**Figure 4.**
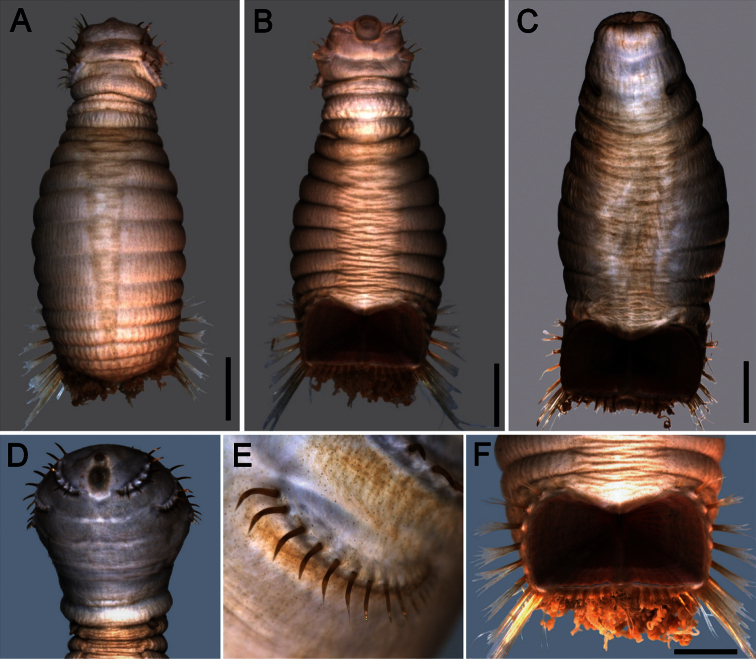
*Sternaspis thalassemoides* Otto, 1821 **A** Neotype (ZMUC POL 2159), dorsal view **B** Same, ventral view **C** Paraneotype, ventral view **D** Another paraneotype, anterior chaetigers, ventral view **E** Same, chaetae of chaetiger 3 **F** Neotype, ventro-caudal shield. Bars: **A** 2.4 mm **B, C** 2.6 mm **F** 1.7 mm.

Body colour off-white or grey in alcohol (Fig. 4A–D); papillae minute, especially behind segment 7 and near shield on dorsal side, or smooth, apparently without papillae. Anterior region often swollen and bulbous, sometimes wider than posterior region, with a constriction at septum between segments 7 and 8 (Fig. 4D). Neotype 14.6 mm long (paraneotypes 11.9–17.0 mm long), 12 mm wide, with about 30 segments.

Prostomium small, without eyespots. Peristomium rounded, flattening at position of the mouth and devoid of any papillae. Mouth circular, completely covered with minute papillae, extends from prostomium to the edge of segment 2 (Fig. 4D).

First three chaetigers with more than 12–14 hooks, bronze with subdistal dark band (Fig. 4E). Genital papillae between segments 7–8 (Fig. 4B–D). Pre-shield region with 7 segments, sometimes bearing small fascicles of fine capillary chaetae.

Ventro-caudal shield with radiating oblique ribs and concentric lines; suture restricted to anterior region (Figs 1B, 4B, C, F). Anterior margins rounded; anterior depression deep; anterior keels not exposed. Lateral margins slightly expanding posteriorly. Fan truncate, not extending beyond posterior corners, crenulated, slightly projected outwardly, especially in larger individuals; median notch shallow.

Marginal chaetal fascicles include 10 lateral ones, chaetae ovally arranged, and six posterior fascicles, chaetae in a slightly curved arrangement. First two lateral fascicles emerge from ventral edge of shield. Lateral fascicles with long hirsute chaetae. Peg chaetae not seen.

Branchiae spirally twisted, abundant, variably eroded (Fig. 4A, B, C, F).

##### Neotype locality.

**Italy**. Naples, Tyrrhenian Sea.

##### Remarks.

*Sternaspis thalassemoides* Otto, 1821 has not been recorded since the late 1800’s and because it is currently regarded as a junior synonym of *Sternaspis scutata* Ranzani, 1817, the type species name disappeared from the literature around the turn of the twentieth century. However, *Sternaspis thalassemoides* is reinstated because it differs from *Sternaspis scutata*, especially regarding the development of the fan; in *Sternaspis thalassemoides* the fan is truncate, entire, reaching the level of the posterolateral corners, whereas it is notched and expanded beyond the posterolateral margins in *Sternaspis scutata*. On the other hand, *Sternaspis assimilis* has been regarded as a junior synonym of *Sternaspis scutata*, but their shields are very different, and *Sternaspis assimilis* shield is more similar to the one of *Sternaspis thalassemoides* because their fan is slightly projected. It would be useful to evaluate the size variation among topotype specimens from the English Channel to ratify or correct this synonymy. Although Otto described the shield as blue-black, the colour varies among most sternaspid species intraspecifically and a few of the 8 individuals had a more typical rust-red coloured shield.

A neotype for *Sternaspis thalassemoides* Otto, 1821 is proposed because this is the type species for *Sternaspis* Otto, 1821 and there are two species in the Mediterranean Sea which have been poorly defined. Further, the lack of type materials and of an adequate description has resulted in confusion such that the species has been regarded as a junior synonym for the other regional species, *Sternaspis scutata* (Ranzani, 1817); the neotype and its description will clarify the taxonomic status of the species (ICZN 1999, Art. 75.3.1–75.3.3). The original material was either not deposited or destroyed, and our queries to collection managers in major European museums concluded that this species has no type material (ICZN 1999, Art. 75.3.4). The original description was brief but the illustrations show a ventro-caudal shield with a straight posterior margin (Otto 1821, fig. 1), which is consistent with the specimen selected as neotype (ICZN 1999, Art. 75.3.5). The proposed neotype was collected in the type locality, Naples (ICZN 1999, Art. 75.3.6), and it has been deposited in the Zoological Museum of the Copenhagen University (ICZN 1999, Art. 75.3.7).

The shield of *Sternaspis thalassemoides* has a posterior margin straight, equal in posterior extension to posterolateral corners resembling *Sternaspis princeps*, *Sternaspis rietschi*, *Sternaspis spinosa* and *Sternaspis thorsoni* sp. n.; however, *Sternaspis spinosa* can be separated from the others because its shield is much wider than long and by having its anterior keels exposed. Further, *Sternaspis thorsoni* can be separated from the others by having more abundant, straw-coloured, delicate introvert hooks, whereas the remaining species have fewer, thicker, darker hooks. Because there are no concentric lines in their shield, *Sternaspis princeps* can be distinguished from *Sternaspis thalassemoides* and *Sternaspis rietschi*. These two species differ because in *Sternaspis thalassemoides* the shield lateral margins are almost straight, not markedly expanded medially, whereas in *Sternaspis rietschi* they are rounded, markedly expanded medially.

#### Sternaspis affinis

Stimpson, 1864, emended

http://species-id.net/wiki/Sternaspis_affinis

Figures 1B 2 5

Sternaspis affinis Stimpson, 1864: 159; von Marenzeller 1890: 5–8, Pl. 1, fig. 7.
Sternaspis fossor : Treadwell 1914: 215; Chamberlin 1919: 405–406; Moore 1923: 21; Berkeley 1930: 69; Berkeley and Berkeley 1941: 19 (list), 51; 1952: 59–60, fig. 123; Hartman 1963: 59, 1969: 351–352, fig. 1; Fauchald 1972: 238–239 (*partim*); Bilyard and Carey 1979: fig. 2, Tab. 2, 1980: 22; Fauchald and Hancock 1981: 35 (*non*Stimpson 1853).
Sternaspis fossor ? : Moore 1908: 358.
Sternaspis scutata : Hartman and Reish 1950: 38; Pettibone 1954: 309–310, fig. 35 a, b (*partim*); Fauchald 1077: 113, fig. 33C, D; Hobson and Banse 1981: 18, 19, 63, Tab. 3, fig. F (*non*Ranzani 1817).

##### Type material.

**Canada, British Columbia, Strait of Georgia**. Neotype (RBCM 005-138-001), and 15 paraneotypes (RBCM 005-138-002), 49°10'47"N, 123°18'02"W, 80 m, 13-III-2003.

**Additional material**. **Canada, British Columbia.** 2 spec. (LACM n2939), Departure Bay, mud and rocks, 18-VII-1940, G.E. & N. MacGinitie, coll. 1 spec. (NHMW 1565), Vancouver Island, 1875. 34 spec. (RBCM 987-254-023), Vancouver Island, southwest of Cape Beale, 48°35'54"N, 125°08'24"W, 104 m, 23-VII-1987. 17 spec. (RBCM 002-148-001), Vancouver Island, Trevor Channel, Helby Island, 48°50'00"N, 125°10'00"W, 19-VI-2002. 1 spec. (RBCM 996-148-004), Vancouver Island, Nanoose Bay, 49°15'30"N, 124°08'30"W, 28 m, 4-VI-1996. 3 spec. (RBCM 991-924-006), Vancouver Island, Saanich Inlet, 48°42'36"N, 123°31'00"W, 60–70 m, 16-II-1987. 24 spec. (RBCM 988-9-032), Dixon Entrance, west of Dundas Island, 54°29'40"N, 131°11'01"W, 143 m, 23-I-1988. Four spec. (RBCM 990-320-043), Vancouver Island, southwest of Nootka Sound, 49°25'14"N, 127°21'55"W, 1000–1166 m, 3-II-1990. **U.S.A., Alaska.** 2 spec. (CAS 151054), Boca de Quadra Inlet, III-1981. 12 spec. (CAS 17805), Gulf of Alaska, Cook Inlet, 59°34'54"N, 151°30'24"W, 99 m, 22-X-1976. 4 spec. (CAS 18987), Chukchi Sea, 67°15'N, 165°25'W, 33 m, 11-IX-1907. 2 spec. (USNM 63142), Gulf of Alaska, 59°51'30"N, 142°06'50"W, 53–100 m, 11-VII-1976. **Washington**. 4 spec. (RBCM 985-474-001), west of Cape Flattery, 48°25'24"N, 125°14'00"W, 168 m, 18-VI-1985. **Oregon**. 8 spec. (USNM 74917), mouth of Columbia River, 91 m, 15-IX-1961. **California**. 20 spec. (ANSP 3315), Monterey Bay, 66 m, 13-V-1904. **Mexico, Gulf of California**. 2 spec. (SIO A838), Isla Angel de Ia Guarda, 562–642 m, 18-I-1968. 16 spec. (SIO A839), Isla Angel de Ia Guarda, 1474 m, 18-I-1968.

##### Description.

**Figure 5.**
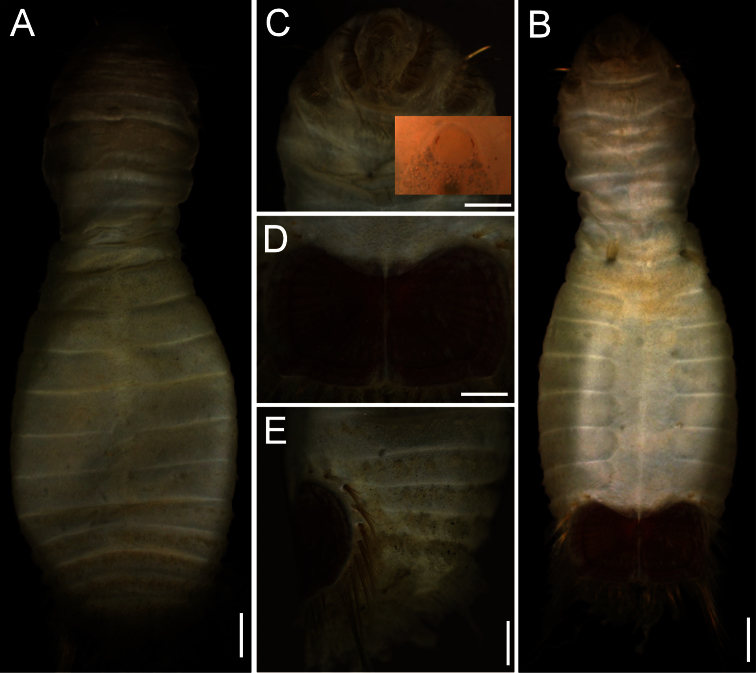
*Sternaspis affinis* Stimpson, 1864, neotype (RBCM 005-138-001) **A** Dorsal view **B** Ventral view **C** Anterior end, frontal view (insert: juvenile, prostomium with eyes) **D** Ventro-caudal shield, frontal view **E** Posterior region, lateral view. Bars: **A** 1 mm **B** 1.1 mm **C** 0.8 mm **D** 0.6 mm **E** 0.7 mm.

Neotype (RBCM 005-138-001), with body cream to light tan, sometimes greyish (Fig. 5A, B). First six segments smooth with a few minute cuticular papillae widely and evenly spaced. Remaining segments more papillate and opaque in appearance. Segments seven and eight slightly more opaque and dense than preceding ones, with stout cuticular papillae especially near genital papillae, some cuticular papillae with small grains of sediment adhered to bases. Body 15.5 mm long, 5.0 mm wide (other specimens up to 22 mm long, 7 mm wide), about 29 segments.

Prostomium hemispherical, opalescent, translucent, sometimes with crescent shaped red eyespots laterally on smaller individuals (Fig. 5C, insert). Peristomium round, without papillae. Mouth oval, covered by papillae, extending from base of prostomium to anterior edge of second segment.

First three chaetigers with 8–14 light bronze, widely separated, slightly falcate introvert hooks per bundle, each with subdistal dark areas (Fig. 5C). Genital papillae protrude ventrolaterally from intersegmental groove between segments 7 and 8.

Pre-shield region with 7 segments, with papillae evenly spaced, slightly denser than on anterior segments, although less so ventrally, and in single rows of clusters of short filaments closer to ventro-caudal shield, especially on dorsal surface, rarely showing delicate short capillary chaetae protruding laterally from body wall.

Ventro-caudal shield with concentric lines, slightly ribbed; suture extended throughout shield (restricted to the anterior region in larger specimens). Anterior margins rounded; anterior depression deep; anterior keels not exposed (Figs 1B, 2, 5B, D). Lateral margins gently rounded (straighter in larger specimens), not expanding posteriorly. Fan truncate, almost straight in juveniles, sometimes with median notch, becoming crenulated in larger specimens.

Marginal chaetal fascicles include 10 lateral ones (Fig. 5E), chaetae ovally arranged, and five posterior fascicles, chaetae in a linear arrangement. Peg chaetae on conical extensions emerging under most prominent oblique rib of the shield. Peg chaetae with stout base in cross section; a small fascicle of delicate capillary chaetae (peg-associated capillary chaetae) between peg chaetae and first fascicle of posterior chaetae.

Branchiae numerous, thick, coiled, slender, long, protruding from two oval plates, separated by a wide angle, on either side of anus. Additional fine, long filamentous papillae extending to lateral and posterior margins of shield.

##### Neotype locality.

British Columbia, Canada, Strait of Georgia.

##### Remarks.

It appears that *Sternaspis affinis* has not been reported since 1875. However, many collections hold specimens collected over the last hundred years of what appears to be the only species present along the northeast Pacific coast of North America, from the Beaufort Sea to California, and into the Gulf of California. These have been labelled either as *Sternaspis scutata* or *Sternaspis fossor*.

The original description by Stimpson is brief and only includes a scant comparison of the cuticle with the Atlantic species, *Sternaspis fossor*. As Stimpson’s description agrees with the characters of the specimens found along the northeast Pacific coast, we propose the emendation above with the designation of a neotype.

The taxonomic status of *Sternaspis affinis* Stimpson, 1864 needs clarification because it has been regarded as a junior synonym of a Northwestern Atlantic species, *Sternaspis fossor* Stimpson, 1853, or of the Mediterranean species, *Sternaspis scutata* (Ranzani, 1817). The proposal of a neotype together with the above description and illustrations will clarify the current situation (ICZN 1999, Art. 75.3.1–75.3.3). The original material was deposited in the Smithsonian and later transferred to Chicago when William Stimpson was appointed director of the local Academy of Sciences in 1866, but they were destroyed in 1871 during the great Chicago fire (http://www.si.edu/oahp/ScientificIllustrators/WStimpson.html; ICZN 1999, Art. 75.3.4). Despite the fact that the original description was brief, *Sternaspis affinis* seems to be the only species living in the type locality region, and we are confident that the neotype corresponds to the species (ICZN 1999, Art. 75.3.5). The proposed neotype was collected in the type locality (ICZN 1999, Art. 75.3.6), and it has been deposited in the Royal British Columbia Museum (ICZN 1999, Art. 75.3.7).

*Sternaspis affinis* resembles *Sternaspis fossor*, *Sternaspis maior* and *Sternaspis islandica* as they all have shields with rounded anterior margins, lateral margins slightly rounded, and posterior margins reaching or slightly expanded beyond the posterolateral corners. However, *Sternaspis islandica* differs by having a very shallow anterior depression, whereas the two other species have deep anterior depressions. The remaining three species differ because in *Sternaspis affinis* and *Sternaspis maior* the radiating ribs and posterior corners are often distinct, whereas they are barely developed, or not at all in *Sternaspis fossor*. Therefore, *Sternaspis affinis* is very similar to *Sternaspis maior* but their main difference lies in the relative development of concentric lines which are distinct in *Sternaspis affinis* and not visible or barely visible in *Sternaspis maior*.

##### Distribution.

Alaska, USA (in the Gulf of Alaska) south along the coast and inland waters to Monterey, California, USA, and into the Gulf of California. This species, identified as *Sternaspis fossor*, has been regarded as one of the most abundant ones along the coast in the East Sound of the San Juan Islands (Weese and Macnab 1930), and along the Washington coast in 95–154 m with sediment having 50–68% mud (Lie and Kisker 1970). Moore (1923: 218) reported two species from Southern California, based upon the number of chaetal fascicles along the shield margins; one with 16 total bundles found in 441–492 m, and the other, smaller in size, with 15 total bundles and collected in sediments at 92–1190 m.

#### Sternaspis africana

Augener, 1918 stat. n.

http://species-id.net/wiki/Sternaspis_africana

Figure 6

Sternaspis fossor var. ** Augener, 1918: 608–613, figs 109–110; Fauvel 1950: 342 (species list).
Sternaspis fossor africana : Petersen 2000: 321, Table 11.1.
Sternaspis scutata var. *africana*: Monro 1930: 179–180; Tebble 1955: 134–135; Kirkegaard 1959: 71–72; Guy 1964: 197; Intes and le Loeuff 1977: 234.
Sternaspis scutata : Jeldes and Lefevere 1959: 32; Rullier 1965: 52–53, fig. 11 (*non*Ranzani 1817).

##### Type material.

Neotype (NHM 1930.10.8.2582), R.V. Discovery Expedition, Angola, St. Paul Loanda, 08°47'S, 13°14'E, in 64–65 m, 4-VIII-1927.

##### Additional material.

**Angola**. 37 spec. (NHM 1930.10.8.2583-90), St. Paul Loanda, 08°47'S, 13°14'E, 64–65 m, 4-VIII-1927. **Cameroon**. 3 spec. (UMML 22.1036), off Malabo Island, R.V. Pillsbury, Cruise 6504, Sta. 259 (03°52'N, 08°54'E), 59 m, 16-V-1965. **Democratic Republic** **of the** **Congo**. 5 spec. (ECOSUR 2648), off Kipundji, 25 m, sand and mud, 25 Aug. 1965, A. Crosnier, coll. **Côte d’Ivoire.** 2 spec. (UMML 22.1041), off Grand Lahou, R. V. Pillsbury, Cruise 6405, Sta. 50 (04°58'N, 05°00'W), 160 m, 31-V-1964. **Gabon**. 1 spec. (NHM 1930.10.8.2581), Cape Lopez, 58–67 m, 8-X-1928. 33 spec. (IRFA-STE 01), Kipundji, 25 m, sand and mud, 25-VIII-1965, A. Crosnier, coll. **Ghana**. 1 spec. (NHM 1953.3.1.489-497), off Accra, Stn 130. 2 spec. (NHM 1953.3.1.489-497), off Accra, Stn 28. Two spec. (NHM 1953.3.1.489-497), off Accra, Stn 47. 1 spec. (NHM 1953.3.1.489-497), off Accra, Stn 59. 2 spec. (NHM 1953.3 .1.489-497), off Accra, Stn 71. **Nigeria**. 1 spec. (UMML 22.1034), off Bonny, R.V. Pillsbury, Cruise 6504, Sta. 254 (03°51'N, 07°10'E), 161 m, 14-V-1965. 1 spec. (UMML 22.1037), off Burutu river mouth, R.V. Pillsbury, Cruise 6504, Sta. 236 (05°19'N, 04°47'E), 114 m, 12-V-1965. 1 spec. (UMML 22.1044), off Burutu river mouth, R.V. Pillsbury, Cruise 6504, Sta. 237 (05°19'N, 04°48'E), 101 m, 14-V-1965.

##### Description.

Neotype (NHM 1930.10.8.2582-90) with body smooth, clean, white, leathery. From segments 6–7, body with minute papillae dense on segments 7 and 8, but evenly spaced in other segments. Well-defined clusters of cuticular papillae in single row starting on segment 8, encircling each segment to posterior end, including last segments opposite ventro-caudal shield. Body up to 20 mm long, 7 mm wide, about 28 segments.

**Figure 6.**
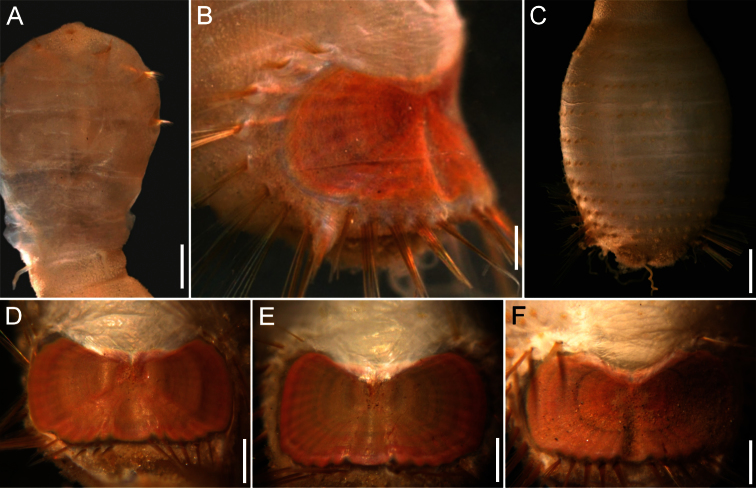
*Sternaspis africana* Augener, 1918 n. status, neotype (NHM 1930.10.8.2582-90) **A** Anterior end, ventral view **B** Posterior end, oblique lateral view **C** Posterior region, dorsal view **D–F** Ventro-caudal shields of three other specimens (IRFA-STE 001). Bars: **A** 2.2 mm **B** 1.7 mm **C** 2.1 mm **D–E** 0.7 mm **F** 0.9 mm.

Prostomium oval, hemispherical, opalescent, translucent (Fig. 6A). Peristomium rounded, raised at the position of mouth and with papillae sparsely covering most of surface. Mouth circular, completely covered by minute papillae, situated halfway between prostomium and anterior border of second segment.

First three chaetigers with 15–20 slender, bronze, slightly falcate hooks in a closely apposed group; hooks without dark areas. One pair of slender translucent genital papillae in intersegmental groove between segments 7 and 8. Pre-shield region with 7 segments, with short couplets of fine capillary chaetae protruding from body wall.

Ventro-caudal shield ribs poorly developed, concentric lines not visible; suture indistinct. Anterior margins angular; anterior depression deep; anterior keels not exposed (Fig. 6B). Lateral margins rounded, expanded medially, reduced posteriorly. Fan barely reaching posterior shield corners, medially projected, denticulated.

Marginal chaetal fascicles include nine lateral ones, chaetae in oval arrangement, and five posterior fascicles, chaetae in a slightly curved arrangement and with each fascicle parallel to next. Peg chaetae long, emerge from an extended fleshy cone; a small fascicle of delicate capillary chaetae emerge from the base of the fleshy cone bearing peg chaetae.

Branchiae mostly eroded, placed on oval, wide branchial plates (Fig. 6C).

##### Variation.

The ventro-caudal shield is medially fused; its fan is slightly projected beyond the posterior margin and its margins are denticulated (Fig. 6D–F). The posterior corners are rounded and never prominent or reaching the fan posterior margin level. Larger specimens may have a median notch and their body papillae are eroded. As originally indicated by Augener (1918: 162–163), the introvert hooks are always thin, abundant and without the subdistal mark which is common in other species in the genus.

##### Neotype locality.

Angola, St. Paul Loanda.

##### Remarks.

Augener (1918) proposed *Sternaspis fossor* var. *africana* for specimens found along the tropical and subtropical Western and southwestern coast of Africa. This species has been regarded as a junior synonym of *Sternaspis scutata* (Ranzani, 1817), a species originally described from the Mediterranean Sea; however, the shields are so different that in order to clarify the status for the Western African species, a neotype is being proposed (ICZN 1999, Art. 75.3.1). The description above and the corresponding illustration characterize the main diagnostic features (ICZN 1999, Art. 75.3.2–75.3.3).

Hermann Augener was a volunteer worker in the Hamburg Museum (CCAM 1938), where he deposited most of his materials; unfortunately, after WWII bombing many type material lots were lost and this included the type series of *Sternaspis fossor* var. *africana*, as confirmed by the museum staff (ICZN 1999, Art. 75.3.4). According to the original description and illustrations by Augener (1918), the ventro-caudal shield has a median fan projection which is unique among the species in the genus; this feature is clearly shown by the neotype and consequently we regard it as consistent with the original type material (ICZN 1999, Art. 75.3.5). Further, the original type localities included a series of places like Senegal, French Guinea, Liberia, Ivory Coast, Gold Coast, Nigeria, French Equatorial Africa, Congo, and Angola, and the proposed neotype was collected in Angola (ICZN 1999, Art. 75.3.6). The neotype has been deposited in the Natural History Museum, London (ICZN 1999, Art. 75.3.7). The original name was introduced as a variety; however, after Art. 45.6.4 (ICZN 1999), the name has subspecific status, as has been listed by Petersen (2000: 321), and consequently we can propose its elevation to species rank.

*Sternaspis africana* Augener, 1918 n. status, resembles *Sternaspis spinosa* because both have shields with deep anterior depressions and markedly expanded lateral shield margins. However, the shield integument is thick in *Sternaspis africana* such that the ribs are barely visible, whereas in *Sternaspis spinosa* the integument is transparent and both ribs and concentric lines are visible. Further, it resembles the only other species having a shield with a denticulate posterior margin: *Sternaspis andamanensis* sp. n., but besides the differences in body papillation which is evident in *Sternaspis africana* and lacking in *Sternaspis andamanensis*, their shields also differ. In *Sternaspis africana* the anterior margins are projected slightly beyond the anterior depression, the fan is not projected medially and there are no lateral notches, whereas in *Sternaspis andamanensis* the anterior margins are markedly projected from the anterior depression, and the fan is markedly projected medially and lateral notches are deep.

##### Distribution.

Western African coast, from Ghana to Angola, 20–70 m.

#### Sternaspis andamanensis sp. n.

urn:lsid:zoobank.org:act:51B7CA16-9014-40D3-BBC1-F7167C26CF03

http://species-id.net/wiki/Sternaspis_andamanensis

Figure 7

##### Type material.

**Andaman Sea, Thailand.** Holotype (ZMUC POL-2157) and two paratypes (ZMUC POL-2158), 7°00'00"S, 99°15'00"E, 45 m, 6-V-1996.

##### Additional material.

**Andaman Sea, Thailand**. 1 spec. (PMBC K1-0S), 7°00'00"S, 99°16'00"E, 41 m, 24-II-1998. **South China Sea, Malaysia**. 1 spec. (AM W 196244), Sarawak, 1982. One spec. (AM W l96245), Sarawak, Bintulu, 5.5 m, 1982.

##### Description.

Holotype (ZMUC POL-2157) with pre-shield and shield regions rounded, much wider than anterior region which is elongate, narrow and bent inwards (Fig. 6A, B). Body papillae few, evenly and widely spaced as filaments over most of surface on segments 1–7; fewer, shorter papillae on segments of shield region. Body up to 8.5 mm long, 5 mm wide, about 28 segments.

Prostomium almost spherical, pale yellow. Peristomium oval, raised at position of mouth. Mouth small, covered by papillae, positioned between prostomium and anterior border of second segment.

**Figure 7.**
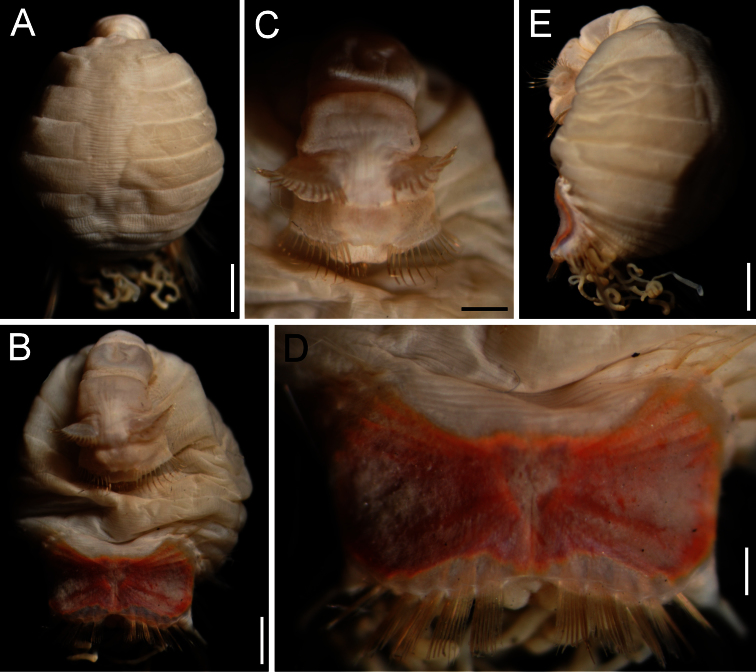
*Sternaspis andamanensis* sp. n., holotype (ZMUC POL-2157) **A** Dorsal view **B** Ventral view, anterior region bent ventrally **C** Same, close-up of chaetigers 2–3 **D** Ventro-caudal shield **E** Lateral view. Bars: **A** 1.2 mm **B** 1 mm **C** 0.6 mm **D** 0.4 mm **E** 1.1 mm (Photos: Jørgen Olesen).

First three chaetigers with 10 larger and up to five smaller flat, bronze, closely associated, falcate hooks per bundle, almost traversing each segment (Fig. 7B, C); hooks with shaft milky, median or subdistal area dark, distal portion light gold. One pair of genital papillae protrude ventrally from intersegmental furrow between segments seven and eight. Pre-shield region with 7 segments, with 2–3 fine capillary chaetae protruding laterally from body wall on some segments.

Ventro-caudal shield ribs barely noticeable, concentric lines not visible; suture poorly defined, apparently extended throughout shield (Fig. 7D). Anterior margins angular; anterior depression deep; anterior keels exposed, with median notch. Lateral margins curved, expanded medially, reduced posteriorly. Fan truncate with two lateral notches and a median, rounded projection, not extended beyond posterior corners, margin denticulated.

Marginal chaetal fascicles include nine lateral ones, chaetae ovally arranged, and five posterior fascicles, chaetae in evenly spaced straight rows. Peg chaetae translucent, lighter in colour than other marginal chaetae, as long as, or longer than posterior fascicles chaetae. Peg chaetae emerge from under shield on a fleshy cone, with a wide base in cross section. Additional fine, short, capillary chaetae next to peg chaetae, medially to first fascicle of posterior shield chaetae.

Branchiae few, stout, tightly coiled (Fig. 7E), protrude from two almost parallel plates.

##### Etymology.

The species name is derived from the Andaman Sea and the suffix indicates it lives in that region.

##### Type locality.

Andaman Sea, Thailand, 45 m.

##### Remarks.

*Sternaspis andamanensis* sp. n. differs in several features from any other species. The arrangement and sparseness of papillae on the cuticle, a narrow anterior region, milky introvert hooks, long and translucent peg chaetae, hourglass-shaped shield, shield chaetae protruding from a translucent band of cuticle around the shield, and posterior chaetae along the shield in an almost continuous row, are all significant differences. The other species having a shield with a denticulate posterior margin is *Sternaspis africana* but besides the differences in body papillation which is evident in *Sternaspis africana* and missing in *Sternaspis andamanensis*, the general shape of the shield differs as well. In *Sternaspis andamanensis* the anterior margins are projected markedly beyond the anterior depression, and the fan is medially markedly projected and the lateral notches are deep, whereas in *Sternaspis africana* the anterior margins are not so projected beyond the anterior depression, and the fan is barely projected medially and there are no lateral notches.

##### Distribution.

Known from two locations: Thailand in the Andaman Sea and Malaysia in the South China Sea, 5–45 m depth.

#### Sternaspis costata

von Marenzeller, 1879, emended

http://species-id.net/wiki/Sternaspis_costata

Figures 1B 8

Sternaspis costata von Marenzeller, 1879: 142–143, Tab. 6, fig. 4; 1890: 5–8, Pl. 1, fig. 5; Sluiter 1890: 108–110;
Sternaspis scutata : Okuda 1936: 151–152, fig. 5; Takahashi 1938: 211, Textfig. 13; Imajima 1961: 94–95, Textfig. 10a–c; Imajima and Hartman 1964: 310-311 (*non*Ranzani 1817).

##### Type material.

**Japan**. Neotype (CMNH ZW-120), Honshu Island, Chiba, Boso Peninsula, 25-V-1995.

##### Additional material.

**Japan**. 2 spec. (ANSP 1051), and 1 spec. (ANSP 1062), off Honshu, 1900. 18 spec. (CMNH ZW-502), Kyushu, Kumamota, Amakusa, Sakitu, 22-VII-1964. 1 spec. (CMNH ZW-514), Honshu Island, Sagami Bay, off Manazuru, 40–70 m. 1 spec. (CMNH ZW-515), Honshu Island, Sagami Bay, off Manazuru, 40–70 m. One spec. (CMNH ZW-617), Kyushu, Kumamota, Amakusa. 1 spec. (CMNH ZW-996), Honshu Island, Sagami Bay, Shimoda, 34°38'53"S, 138°57'07"E, 40 m. 8 spec. (NHMW 1568), Honshu Island, Nagoya Bay, 1877. **Sakhalin Island, Russia.** 5 spec. (ZIRAS 43188), Aniva Bay, RV Toporok, Sta. 47, 46°20.8'N, 142°34.8'E, 48 m, 21 Sep. 1947. **Philippines**. 1 spec. (AM W 27162), west coast of Marinduque Island, 13°30'00"S, 121°30'00"E.

##### Description.

**Figure 8.**
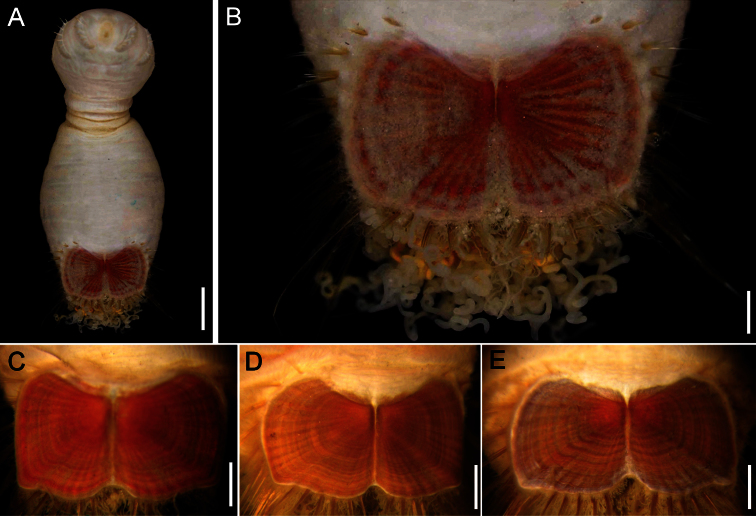
*Sternaspis costata* von Marenzeller, 1879, emended, neotype (CMNH ZW-120) **A** Complete, ventral view **B** Ventro-caudal shield **C–F** Sakhalin Island specimens (ZIRAS 43188), ventro-caudal shields. Bars: **A** 2 mm **B** 0.5 mm **C** 0.9 mm **D** 1.4 mm **E** 1.5 mm (Photos D–E: Eijiroh Nishi).

Neotype (CMNH ZW-120) with body colour creamy white to yellow-white, sometimes more grey, with first six segments lighter, becoming darker when dried out (Fig. 8A). Cuticle mostly with short filamentous papillae, somewhat longer on segments seven and eight. Rows of clustered filamentous papillae usually in two loosely arranged, lateral rows per segment, more noticeable on posterior segments dorsal to ventro-caudal shield. Body up to 22 mm long, 10 mm wide, about 29 segments.

Prostomium small, hemispherical, slightly opalescent. Peristomium rounded, raised at mouth, with some papillae between mouth and prostomium. Mouth densely papillate, slightly oval, positioned halfway between prostomium and anterior edge of segment 2.

First three chaetigers with 10 bronze, slightly falcate, introvert hooks with about another five smaller hooks ventral to larger hooks. Hooks widely separated (widely apposed), with subdistal dark areas. One pair of genital papillae protrude ventrally from intersegmental furrow between segments 7 and 8. Pre-shield region with 7 segments, with small fascicles of fine short capillary chaetae laterally in some specimens.

Ventro-caudal shield dark orange, often covered with sediment; ribs and concentric lines visible; suture extended throughout the shield (Figs 1B, 8B–E). Anterior margins rounded; anterior depression shallow; anterior keels not exposed. Lateral margins rounded, expanded posteriorly. Fan slightly projected posteriorly, markedly notched medially.

Marginal chaetal fascicles include 10 lateral ones, chaetae in a narrow oval arrangement, and five posterior fascicles in an offset linear arrangement; chaetae curving towards midline. Peg chaetae long, with a narrow base in cross section, emerge from cuticle almost at same level as margin of shield. Two additional groups of delicate chaetae between peg chaetae and first bundle of posterior shield chaetae.

Branchiae numerous, coiled and protrude from two plates widely separated dorsally.

##### Variation.

The specimens from the Sakhalin Island (Fig. 8C–E) show that the posterior median notch is always wide, but there are some changes with size. For example, from smaller to larger specimens, the anterior corners become less prominent whereas the diagonal rib and the fan ribs become more prominent. The relative posterior extension of the fan tends to become reduced and in even larger specimens, it may disappear completely.

##### Neotype locality.

Honshu Island, Chiba, Boso Peninsula, Japan.

##### Remarks.

*Sternaspis costata* von Marenzeller, 1879 has a rather peculiar nomenclatural history because it was the same author who proposed the species who later concluded (von Marenzeller 1890) it was a junior synonym of another species, *Sternaspis scutata* (Ranzani, 1817) originally described from the Mediterranean Sea. As stated below, these two species are different and the Japanese species must be clarified; consequently we propose and describe a neotype and provide illustrations for its diagnostic features (ICZN 1999, Art. 75.3.1–75.3.3). Emil von Marenzeller worked in the Vienna Museum and was in charge of several invertebrate groups, including polychaetes; however, because he changed his mind about his own species, he might have sent away the apparently discarded type materials or destroyed them during dissection because Dr. Helmut Sattmann has informed us that there is no type material for this species (ICZN 1999, Art. 75.3.4). Von Marenzeller made only two figures and a detailed description to emphasize that his new species differed by the relative rib development, and his illustration shows that the fan is truncate with a deep median notch and that the posterior shield corners are well-developed; these same features are shown by the neotype such that we regard it as consistent with the original description and illustrations (ICZN 1999, Art. 75.3.5). The original type locality was Miya Bay, south of Nagoya, Honshu Islands, Eastern Japan and the neotype locality is the Boso Peninsula, Chiba, Eastern Japan, about 300 km away but along the same coast. Despite the fact that these two localities are not contiguous, they are very close to each other (ICZN 1999, Art. 75.3.6), although there was no indication about depth or habitat for the original materials. The neotype has been deposited in the Coastal Branch of Natural History Museum and Insitute, Chiba, Japan (ICZN 1999, Art. 75.3.7).

Despite von Marenzeller’s detailed description of the ventro-caudal shield of *Sternaspis costata*, and especially because he later regarded it as a junior synonym of *Sternaspis scutata*, it was not recorded under the original name. There is no close resemblance between these two species because they markedly differ in their shields. In *Sternaspis costata* the anterior margins are rounded, the lateral margins expanded medially, the posterior corners are angular, well-defined, and the fan is markedly notched medially. On the contrary, in *Sternaspis scutata* the anterior margins are truncate, the lateral margins are straight, barely expanded, the posterior corners are rounded, poorly defined, and the fan is barely notched medially, and projected beyond the posterior corners. *Sternaspis costata* is unique among the species in the genus because its shield fan is reduced along its median line, especially in larger specimens, such that the lateral fan portions are longer, reaching the posterior corners, but the median portion is very short, as if having a wide, deep median notch.

##### Distribution.

Southern Sakhalin Island (Russia), Japan, and the Philippines, 20–70 m depth. The record for estuarine environments in India (Southern 1921: 649–651, Pl. 20, fig. 5a, b) is questionable; the illustration resembles the species but there are some subtle differences. Therefore, we are doubtful about the distribution extending to estuarine waters in the Bay of Bengal.

#### Sternaspis fossor

Stimpson, 1853, restricted

http://species-id.net/wiki/Sternaspis_fossor

Figures 1B 9

Sternaspis fossor Stimpson, 1853: 29, fig. 19; Verrill 1873: 606, Pl. 14, fig. 74; Webster and Benedict 1884: 725, 1887: 132; von Marenzeller 1890: 5–8, Pl. 1, fig. 4A–B; Moore 1909: 144; Hartman 1944: 82, Pl. 33, fig. 15; Hartman 1965: 192.
Sternaspis scutata : Pettibone 1954: 309–310, fig. 35 a, b (*partim*, *non*Ranzani 1817)

##### Type material.

**Northwestern Atlantic Ocean, Canada**. Neotype (USNM 15543), 88 km E Cape Sable, Nova Scotia, 153 m, mud, 6 Oct. 1909, O. Bryant, coll.

##### Additional material.

**Canada, Brunswick**. 1 spec. (HMCS 9953670), Bocabec Bay, 45°10'N, 67°02'W, 22 m, 20-XII-1976. 1 spec. (HMCS 9953671), L’Etang Estuary, 45°04'30"N, 66°47'39"W, 20-VIII-1975. 37 spec. (HMCS 9953672), Letite Passage, 45°03'N, 66°55'W, 73 m (in codfish stomach), 7-V-1976. 5 spec. (HMCS 9953673), Passamaquoddy Bay, Loring Cove, 45°06'N, 66°59'W, 27–34 m, 22-V-1973. 12 spec. (HMCS 9953676), Bocabec Bay, 45°10'N, 67°02'W, 3-III-1977. 1 spec. (HMCS 9953677), Passamaquoddy Bay (Wolves-Lepreau), 1966. 4 spec. (USNM 7872), East of Grand Manan, 108 m, mud, 1872. **U.S.A.** Three spec. (ANSP 1247), off Newport, Rhode Island.

##### Description.

**Figure 9.**
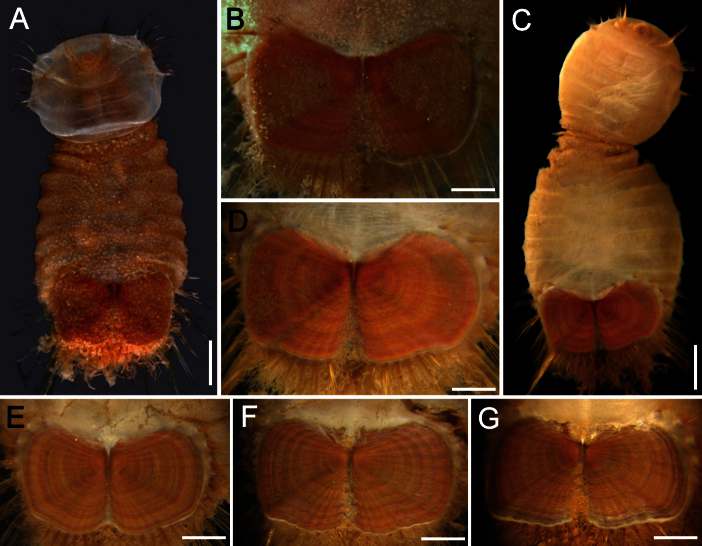
*Sternaspis fossor* Stimpson, 1853 **A** Juvenile (HMCS 9953670), anterior end exposed, ventral view **B** Another specimen (HMCS 9953671), ventro-caudal shield, frontal view **C** Neotype (USNM 15543), anterior end exposed, ventral view **D** Same, ventro-caudal shield, frontal view **E–F** Ventro-caudal shields of three other specimens (USNM 7872). Bars: **A**, **F** 1 mm **B** 0.6 mm **C**, **E** 0.8 mm **D** 0.7 mm **G** 1.2 mm.

Neotype (USNM 15543) complete, most body papillae eroded but transverse rows still noticeable; introvert exposed (Fig. 9C); 9.7 mm long, 3.5 mm wide, 31 segments. Body colour in alcohol often tan to light brown, sometimes ashen or cinereous (Fig. 9A, C). Cuticular papillae evenly distributed over most of the body especially posteriorly, starting at segment 8. Single transverse dorsal rows of clusters of papillae per segment, especially towards posterior end. First seven segments usually much cleaner and translucent, especially in smaller individuals. Body up to 15 mm long, 8 mm wide, about 31 segments.

Prostomium hemispherical, opalescent, without eyespots, minutely granular in appearance. Peristomium rounded, without papillae, slightly raised near mouth. Mouth slightly oval, completely covered by papillae, extends from prostomium almost to edge of segment 2.

First three chaetigers with 6–12 bronze, widely separated, slightly falcate hooks per ramus, with subdistal dark areas, transparent in juveniles, opaque in larger specimens (Fig. 9C). Genital papillae protrude ventrally from intersegmental groove between segments 7 and 8. Pre-shield region with 7 segments, with small, short fascicles of fine capillary chaetae protruding laterally from body wall in some small specimens.

Ventro-caudal shield ribbed; juveniles with few concentric lines darker than the background shield colour, often covered by sediment (Fig. 9B), concentric bands better defined in larger specimens (Fig. 9D); suture extended throughout shield. Anterior margins rounded; anterior depression deep; anterior keels not exposed. Lateral margins straight in smaller specimens, curved in larger specimens, expanding posteriorly. Fan slightly projected beyond posterior corners, smooth in juveniles, crenulated in larger specimens, with a median shallow notch (Figs 1B, 9B).

Marginal chaetal fascicles include 10 lateral ones, chaetae in an oval arrangement, and 6–7 posterior fascicles, chaetae arranged in an approximately ventro-dorsal line. Lateral chaetae light bronze proximally along the shafts, grading to almost clear at the distal ends. Peg chaetae short, often obscured by adhered sediment or filamentous papillae among bases of chaetae. Additional short delicate capillary chaetae between peg chaetae and first posterior fascicle of shield chaetae.

Branchiae stout, coiled, protruding from two oval, obliquely set plates, one on either side of anus. Many long filamentous interbranchial papillae with sediment particles attached.

##### Variation.

The ventro-caudal shield is covered with sediment which is adhered to thin papillae in smaller specimens. Larger specimens have sediment particles less firmly adhered and can be brushed off. The pigmentation pattern is banded with concentric lines well-defined but ribs barely prominent; the fan is slightly projected and markedly cleft (Fig. 9E–F, G), and the posterior margin is smooth in smaller specimens becoming slightly crenulated in larger specimens.

##### Remarks.

The taxonomic status of *Sternaspis fossor* Stimpson, 1853 requires clarification because it has been regarded as a widely distributed species, or has been taken either as a senior synonym of the Northwestern Pacific species, *Sternaspis affinis* Stimpson, 1864, or as junior synonym for the Mediterranean species, *Sternaspis scutata* (Ranzani, 1817). In order to clarify this situation, a neotype has been proposed together with the above description and illustrations (ICZN 1999, Art. 75.3.1–75.3.3). As for *Sternaspis affinis* (see above), Stimpson’s original material was destroyed during the great Chicago fire in 1871. Despite the fact that the original description was brief, *Sternaspis fossor* is apparently the only species living in the type locality region, and we are confident that the neotype corresponds to the species (ICZN 1999, Art. 75.3.5). The above proposed neotype was collected nearby the type locality, Grand Manan Channel (ICZN 1999, Art. 75.3.6), although there were no details about depth or sediment type. The neotype has been deposited in National Museum of Natural History (ICZN 1999, Art. 75.3.7).

*Sternaspis fossor* resembles *Sternaspis affinis*, *Sternaspis islandica* and *Sternaspis maior* because their shields are provided with rounded anterior margins, the lateral margins are slightly rounded, and the posterior margins are slightly expanded beyond the posterolateral corners. However, *Sternaspis islandica* differs by having a very shallow anterior depression, whereas the two other species have a deeper anterior depression. The three other species differ especially in the ornamentation of the shield surface because in *Sternaspis fossor* the radiating ribs and posterolateral corners are poorly developed, barely visible, whereas in *Sternaspis affinis* and *Sternaspis maior* they are often distinct.

##### Distribution.

Northwestern Atlantic Ocean, from Canada to the northeastern United States coast, in 20–153 m. Other records (Augener 1906: 191, Wesenberg-Lund 1962: 142) need confirmation. The distribution of the true *Sternaspis fossor* is probably much less extensive than previously thought, and may be confined to the east coast of Canada and northeastern coast of the United States.

#### Sternaspis islandica

Malmgren, 1867

http://species-id.net/wiki/Sternaspis_islandica

Figure 10

Sternaspis islandica Malmgren, 1867: 196–197, Pl. 14, fig. 85A–D1.

##### Type material

**Iceland**. Eight syntypes (SMNH 5135), Berufjord, 64°48'N, 14°30'W, 27–55 m, 1857.

##### Additional material

**Iceland**. 1 spec. (ZMUC “Ingolf 129”), N off Flateyri, 66°35'N, 23°47'W, 220 m, 3-VIII-1896. 4 spec. (ZMUC Dana 6131), near Keflavik, 64°02'N, 22°31'W, 33 m, 22-VII-1939. 9 spec. (ZMUC 51), Faxelfjord, 3m, 17-VIII-1901. 31 spec. (ZMUC), Faxelfjord, Hammisvork. Seven spec. (ZMUC), Faxefj., 2 km N off Keflark, 6 m, 7-VIII-1901. 1 spec. (ZMUC), Faxefjord, 7-9 m, 7-VII-1899. 50 spec. (IMNH 2526), NW off Flateyri, 66°36'20"N, 23°58'37"W, 226 m, 13-VII-1993. 9 spec. (IMNH 2568), N off Flateyri, 66°55'18"N, 23°30'58"W, 196 m, 15-VII-1993. 5 spec. (IMNH 2994), SE off Hofn, 63°45'36"N, 14°50'36"W, 216 m, 5-VII-1997. 2 spec. (IMNH 3062), E off Hofn, 63°59'32"N, 14°08'49"W, 218 m, 10-VII-1997. 9 spec. (MNHN 451), off Northern Iceland, R.V. Pour-quoi pas?, Stat. 24 (66°37'N, 23°50'W), 160 m, 25 Jul. 1912**. Denmark, Faroe Islands**. 3 spec. (ZMUC), Aknoeyr, 4 m, 8-IX-1901.

##### Description

**Figure 10.**
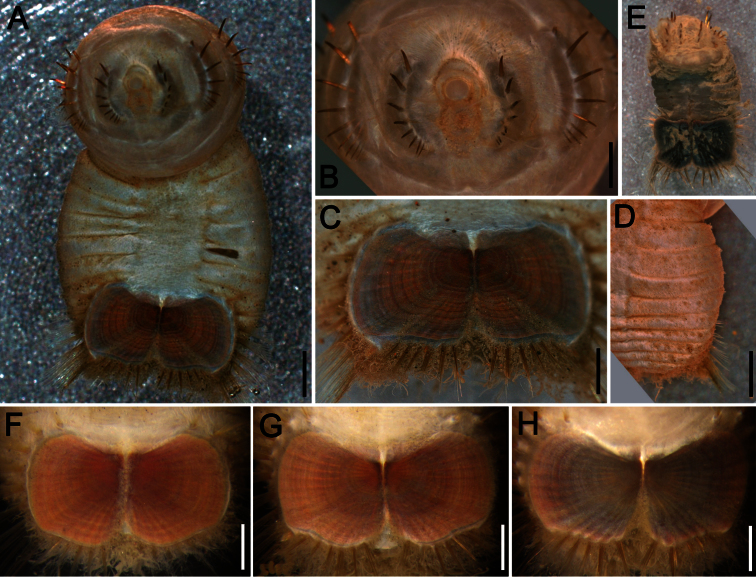
*Sternaspis islandica* Malmgren, 1867, syntypes (SMNH 5135) **A** Complete syntype, ventral view **B** Same, anterior end, frontal view **C** Same, ventro-caudal shield **D** Same, posterior region, dorsal view **E** Non-type specimen (ZMUC), with darker, blackish shield **F–H** Ventro-caudal shield of other non-type specimens (MNHN). Bars: **A** 1.2 mm **B** 1.4 mm **C, D** 1.3 mm **F** 0.7 mm **G–H** 1 mm.

(Based on best syntype). Body with first six segments smooth, pale, without cuticular papillae (Fig. 10A). Segments seven and eight with many small cuticular papillae, decreasing in density ventrally on remaining posterior segments, more numerous on the dorsal surface opposite the shield. Single rows of clusters of longer filamentous cuticular papillae present especially dorsally near ventro-caudal shield (Fig. 10D). Body 10 mm long, 5.5 mm wide, 30 segments.

Prostomium hemispherical, opalescent, finely granular. Peristomium round, flattened at mouth, without papillae. Mouth oval, covered by papillae, extends from edge of prostomium to the anterior border of segment 2 (Fig. 10B).

First three chaetigers with six to 12–14 bronze, slightly falcate introvert hooks, each with subdistal dark areas. Genital papillae protrude ventrally from intersegmental groove between segments 7 and 8. Pre-shield region with 7 segmentswithout chaetae.

Ventro-caudal shield rust red, with fine oblique ribs, and regularly spaced concentric lines; suture extended throughout shield (Fig. 10C); dried out syntypes with a darker, blackish shield (Fig. 10E). Anterior margins rounded; anterior depression deep; anterior keels not exposed. Lateral margins expanded posteriorly. Fan truncate, margin smooth, slightly sigmoid, with two shallow lateral, and median deeper notches.

Marginal chaetal fascicles include 10 lateral ones, ovally arranged, and six posterior fascicles, also in oval arrangement. Chaetae of fascicles nine and ten are about 1.5 x the length of the remaining lateral fascicles. Peg chaetae short, broad, oval in cross section at the base. Additional delicate capillary chaetae between peg chaetae and first posterior fascicle of shield chaetae.

Branchiae coiled filaments, emerge from two branchial plates, oriented close to parallel. Few long filamentous interbranchial papillae among branchiae.

##### Variation.

Most syntypes with dark brown body walls, probably after some dehydratation and variably damaged; one broken into two parts, others with shield completely detached or one plate dislodged. Other specimens (MNHN 451) show that shields become progressively darker and that their ribs are progressively better defined as body grows; at the same time, the fan can be slightly to markedly projected beyond the level of the posterolateral corners.

##### Remarks.

*Sternaspis islandica* Malmgren, 1867 does not appear in the literature except in some faunal accounts where the name was considered a junior synonym of *Sternaspis scutata*, such as Fauvel (1927), Wesenberg-Lund (1950, 1951), and Ushakov (1955).

*Sternaspis islandica* and *Sternaspis rietschi* Caullery, 1944 are very similar because their ventro-caudal shields have shallow anterior depressions, and their concentric lines are more visible than the radial ribs. However, these two species differ because in *Sternaspis islandica* the posterior shield corners are projected, whereas in *Sternaspis rietschi* they are not prominent at all. A lectotype was not selected because of the general condition of the type materials.

##### Distribution.

Apparently restricted to the Norwegian Sea and Northeast Atlantic Ocean around Iceland and the Faroe Islands, 7–226 m depth.

#### Sternaspis maior

Chamberlin, 1919

http://species-id.net/wiki/Sternaspis_maior

Figure 11A–G

Sternaspis maior Chamberlin, 1919: 406-407, Pl. 78, fig. 10.
Sternaspis fossor : Fauchald 1972: 238–239, Méndez 2007: 609, 614, 616 fig. 7 (*partim, non*Stimpson 1853).

##### Type material.

**Eastern Tropical Pacific, Gulf of California**. Neotype (UNAM 7882), RV El Puma, Crucero Talud V, Sta. 25 (24°52'N, 108°58'W), off Isla Altamura, Sinaloa, 830 m, 16-XII-2001, N. Méndez, coll. 1 paraneotype (UNAM 7881), RV El Puma, Crucero Talud V, Sta. 18 (24°15'N, 108°17'W), off Ensenada del Pabellón, Sinaloa, 965 m, 15-XII-2000, N. Méndez, coll. 1 paraneotype (UNAM 0000), RV El Puma, Crucero Talud XIV, Sta. 13 (28°31'34"N, 112°17'43"W), dredge, 180-182 m, 8-IV-0000, B. Yáñez, coll.

##### Description.

**Figure 11.**
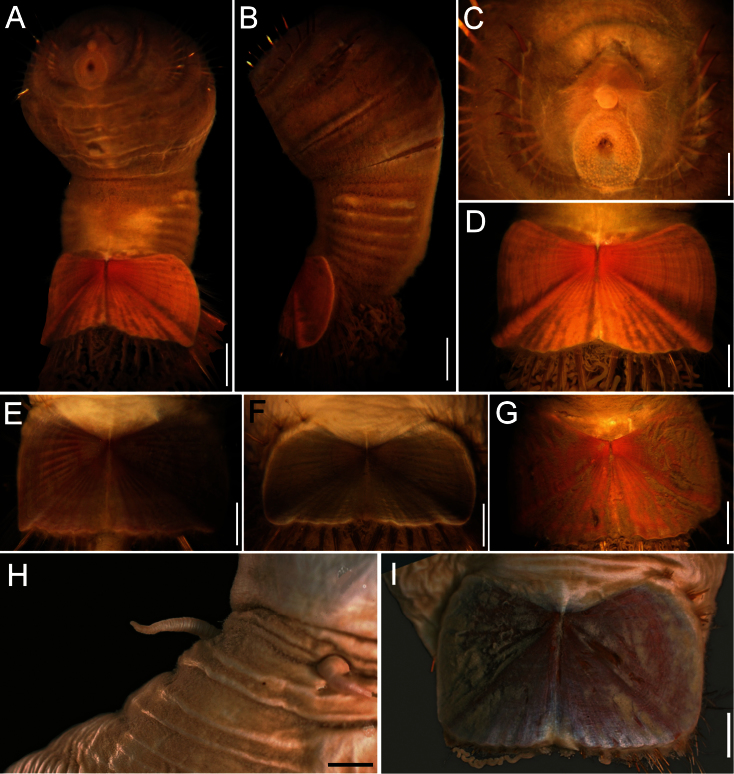
*Sternaspis maior* Chamberlin, 1919 **A** Neotype (UNAM 7882), ventral view **B** Same, lateral view **C** Same, anterior end, frontal view **D** Same, ventro-caudal shield **E** Paraneotype (UNAM Sta. 13), ventro-caudal shield **F** Paraneotype (UNAM Sta. 13, OH), ventro-caudal shield **G** Paraneotype (UNAM 7881), ventro-caudal shield. *Sternaspis princeps* Selenka, 1885, syntypes (NHM 1885.12.3.1) **H** Larger syntype, median region showing gonopodial lobes **I** Smaller syntype, ventro-caudal shield, frontal view. Bars: **A** 1.9 mm **B** 2 mm **C** 1 mm **D** 1.4 mm **E** 1.3 mm **F** 1.5 mm **G** 2.5 mm **H, I** 1.2 mm.

Neotype (UNAM 7882), with body browinish, paler without the papillar layer (Fig. 11A, B). Introvert expanded, markedly wider than abdomen, covered with abundant small papillae. Abdomen with abundant, homogeneously distributed papillae. Body 17 mm long, 6 mm wide (complete paraneotypes 19.5–20.0 mm long, 7–10 mm wide), about 29 segments.

Prostomium hemispherical, paler than surrounding areas (Fig. 11C). Peristomium round, without papillae. Mouth oval, covered by papillae, restricted to a circular region around the mouth.

First three chaetigers with 12–14 golden, widely separated, falcate introvert hooks per bundle, each with subdistal dark areas (Fig. 11B, C). Genital papillae lost, eroded from the intersegmental groove between segments 7 and 8.

Pre-shield region with 7 segments, with papillae abundant, evenly distributed. No capillary chaetae seen.

Ventro-caudal shield with ribs, but no concentric lines; suture restricted to anterior region. Anterior margins rounded; anterior depression shallow; anterior keels not exposed (Fig. 11A, D). Lateral margins gently rounded, expanded posteriorly. Fan truncate, not extended beyond posterior shield corners, with a median notch, crenulated.

Marginal chaetal fascicles include 10 lateral ones (Fig. 11B, D), chaetae ovally arranged and 8 posterior fascicles, chaetae in linear arrangement. Peg chaetae on conical extensions emerging under shield corners. Peg chaetae with stout base in cross section; a small fascicle of delicate capillary chaetae (peg-associated capillary chaetae) between peg chaetae and first fascicle of posterior chaetae.

Branchiae numerous, thick, coiled, slender, long, protruding from two oval plates, separated by a wide angle, on either side of the anus. Additional fine, long filamentous papillae extending along the posterior margin of the shield.

##### Variation.

The shield varies from dark reddish to orange (Fig. 11E–G) although their relative width varies depending on how heavily contracted the abdomen is, and how this contraction bends the lateral plates dorsally resulting in an apparently narrower looking shield. The main radial rib is very prominent, the fan is crenulated but it may be truncate, barely reaching the posterior corners (Fig. 11E, F), or projected beyond this corners (Fig. 11D, G).

##### Neotype locality.

Off Isla Altamura, Sinaloa, Gulf of California, 830 m depth.

##### Remarks.

*Sternaspis maior* Chamberlin, 1919 was very briefly described and the main distinguishing features were based upon the shield. Judging from the dimensions of the ventro-caudal shield (7 mm long, 15 mm wide), the original specimen must have been very large, but perhaps his specimen was severely damaged and only the shield could be characterized.

It is noteworthy that Chamberlin and Augener (1918, for *Sternaspis africana*, see above) almost simultaneously based their descriptions on schematic shield illustrations. Both illustrations indicate significant resemblances to the specific shields shape and ornamentations of *Sternaspis maior*. In both species, the shield was illustrated as having no concentric lines; for *Sternaspis maior*, the anterior depression had large keels, the main radial rib is quite distinct, and the fan has a median notch. These features are all present on the neotype such that we are confident we found the same species, and that this species is distinct. Thus, in order to clarify its taxonomic status (ICZN 1999, Art. 75.3.1), a neotype has been selected, described and its diagnostic features have been illustrated (ICZN 1999, Art. 75.3.2–75.3.3). Hartman (1938: 3) emphasized that many type specimens which were supposedly deposited in Harvard, were not found in the collections and this includes the type materials of *Sternaspis maior*, such that we can conclude there is no type material available (ICZN 1999, Art. 75.3.4). We regard the neotype as conspecific with the specimen described in the original description (ICZN 1999, Art. 75.3.5). The original type locality was from the Gulf of California, south of Guaymas, Sonora (27°39'40"N, 111°00'30"W), 1143 m, and the proposed neotype was collected in a nearby locality, along the eastern Gulf of California coast, and in similar depths to the original material (ICZN 1999, Art. 75.3.6). The neotype and paraneotypes are deposited in the Marine Benthic Invertebrates Reference Collection of the Mazatlán Academic Unit, UNAM (ICZN 1999, Art. 75.3.7).

*Sternaspis maior* resembles *Sternaspis affinis* because both species have shields with round anterior margins, fan projected beyond the level of the posterior corners and with a median notch. The main difference relates to the presence of concentric lines which are barely visible in *Sternaspis maior* and distinct in *Sternaspis affinis*.

##### Distribution.

Central part of the Gulf of California, México, in soft bottoms at 180–965 m, but the original material was collected at 1143 m.

#### Sternaspis princeps

Selenka, 1885

http://species-id.net/wiki/Sternaspis_princeps

Figure 11H, I

Sternaspis princeps Selenka, 1885: 5–6, Pl. 1, fig. 1.

##### Type material.

**South Pacific Ocean**. **New Zealand**. Two syntypes (NHM 1885.12.3.1), R.V. Challenger, North Island, NE off Gisborne, 37°34'S, 179°22'E, 1274 m, 10-VII-1874.

##### Description.

Syntypes (NHM 1885.12.3.1) body smooth, except for longitudinal wrinkles starting on segment eight, probably an artefact of fixation and/or preservation process (Fig. 11H). Colour white, slightly opalescent, dirty white on posterior segments. Cuticle covered by minute papillae, especially on segments seven and eight and the segments near ventro-caudal shield. Body up to 29 mm long, 11 mm wide, 30 segments.

Prostomium hemispherical, opalescent, light yellow in colour. Peristomium rounded, raised at position of mouth and without papillae. Mouth oval, covered by minute papillae, extends from edge of second segment halfway to the border of prostomium.

First three chaetigers with about 10–15 bronze, widely separated, slightly falcate introvert hooks, each with subdistal, narrow dark areas. Genital papillae protrude ventrally from intersegmental groove between segments 7 and 8 (Fig. 11I). Pre-shield region with 7 segments, sometimes with row of small, short fascicles of fine capillary chaetae, barely protruding from body wall laterally.

Ventro-caudal shield surface almost flat. Shield surface faintly ribbed with one larger oblique rib; suture indistinct, barely defined anteriorly, poorly defined posteriorly (Fig. 11I); larger syntype with faint concentric lines, smaller individual with more distinct concentric lines. Anterior margins rounded; anterior depression deep; anterior keels not exposed. Lateral margins straight, barely expanded posteriorly. Fan truncate, margin crenulated, with shallow median notch.

Marginal chaetal fascicles include ten lateral ones, and six posterior fascicles; all chaetae broken on both syntypes, except for first two lateral fascicles. Peg chaetae present as stubs. Additional chaetae damaged.

Branchiae lost; branchial plates visible, oriented close to parallel with respect to each other.

##### Remarks.

Selenka (1885) indicated a shallow furrow running along the middle of the ventral surface, dividing each half into a larger anterior triangle and a smaller posterior triangle. Although he did not indicate this specifically, he was probably referring to the anterolateral and posterior portions of the shield. He also counted 40 tufts of chaetae along the margins of the shield. If the secondary groups of chaetae, such as the delicate fascicles at the posterolateral edges are included, there are still only 34. Because one syntype is very large, and chaetal fascicles may be irregularly broken, he might have inadvertently counted a few of the fascicles more than once.

There are five species having shields with straight posterior margins: *Sternaspis princeps*, *Sternaspis rietschi*, *Sternaspis spinosa*, *Sternaspis thalassemoides* and *Sternaspis thorsoni* sp. n. *Sternaspis princeps* is most similar to *Sternaspis thalassemoides* because both have deep anterior depressions and rounded anterior margins. However, they differ because in *Sternaspis princeps* only the larger, radial rib is more or less visible, but concentric lines are not, whereas in *Sternaspis thalassemoides* the shield has radial ribs and concentric lines. An additional difference is that in *Sternaspis princeps* the shield anterior keels are exposed whereas they are covered in *Sternaspis thalassemoides*.

##### Distribution.

Only known from the type locality, off North Island, New Zealand, about 1274 m depth.

#### Sternaspis rietschi

Caullery, 1944

http://species-id.net/wiki/Sternaspis_rietschi

Figure 12

Sternaspis rietschi Caullery, 1944: 68–70, fig. 54a–c; Bleeker and van der Spoel 1992: 159.

##### Type material.

**Indonesia**. Holotype (ZMA 1500), west of Wokam Island, 5°46'S, 134°00'E, 1788 m, 1899–1900, Stn. 271.

##### Description.

**Figure 12.**
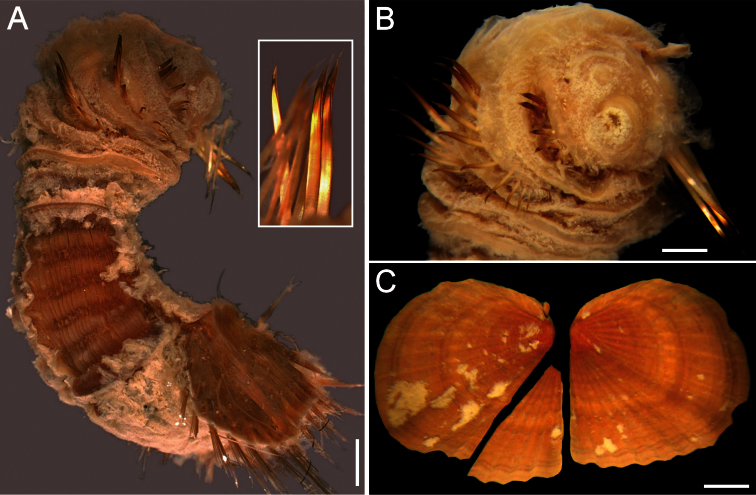
*Sternaspis rietschi* Caullery, 1944, holotype (ZMA 1500) **A** Complete specimen, oblique, lateral view (insert: hooks from chaetiger 3) **B** Same, anterior end, frontal view **C** Ventro-caudal shield, pieces previously broken, frontal view. Bars: **A** 0.5 mm **B**: 1 mm **C** 1.3 mm.

Holotype (ZMA 1500) damaged; integument removed from several body regions; ventro-caudal shield previously removed. Introvert without integument over the first chaetigers, abdomen with integument and body wall broken laterally. Body papillae difficult to determine due to the poor condition of holotype (Fig. 12A). Body 18 mm long, 5 mm wide, about 29 segments.

Prostomium hemispherical, opaque, tan in colour. Peristomium rounded, slightly raised at position of the mouth, without papillae. Mouth oval, covered by papillae, extends from anterior edge of segment 2 almost to prostomium (Fig. 12B).

First three chaetigers with about six to ten large, and five or more smaller, bronze, widely separated, slightly falcate hooks; each with subdistal darker area (Fig. 12A, B). Genital papillae flattened, short protrude ventrally from intersegmental groove between segments 7 and 8. Pre-shield region with 7 segments, without fine capillary chaetae.

Ventro-caudal shield previously removed, broken into three pieces; surface pale brown; ribs barely visible, concentric lines visible; suture probably indistinct (Fig. 12C). Anterior margins rounded; anterior depression shallow; anterior keels probably not exposed. Lateral margins medially expanded, reduced posteriorly. Fan truncate, margin crenulated, median notch shallow, or indistinct (shield plates previously separated).

Marginal shield chaetal fascicles include ten lateral ones, chaetal pattern unknown, and five posterior fascicles, chaetal pattern unknown. Peg chaetae short, with a broad base in cross section, emerge from cuticle on a slightly raised mound. Additional chaetae delicate, between peg chaetae and first bundle of posterior chaetae.

Branchiae lost; nature of branchial plates not determined.

##### Remarks.

The holotype is in poor condition with most of the cuticle missing, exposing the musculature below. It is a large specimen which exaggerates some of the features such as those of the shield and colouring of the introvert hooks. Caullery reported 16 chaetal fascicles in total with 8 located posteriorly; however, because the shield was separated, it appears he counted the groups of chaetae as they appeared under the shield. Further it would have been difficult to determine correctly whether the delicate fine group is part of posterior or lateral fascicles.

The shield of *Sternaspisrietschi* has a posterior margin straight, at same level as margin of shield resembling *Sternaspis princeps*, *Sternaspis spinosa*, *Sternaspis thalassemoides* and *Sternaspis thorsoni* sp. n. As indicated above, *Sternaspis spinosa* differs from the others in that its shield is much wider than long and by having exposed its anterior keels. Further, *Sternaspis thorsoni* has more abundant, straw-coloured, delicate introvert hooks, whereas the remaining species have fewer, thicker, darker hooks. Also, there are no concentric lines on the shield of *Sternaspis princeps* in contrast to *Sternaspis thalassemoides* and *Sternaspis rietschi*. These two species differ because in *Sternaspis rietschi* the shield lateral margins are rounded, markedly expanded medially, whereas in *Sternaspis thalassemoides* they are rather straight, not markedly expanded medially.

##### Distribution.

Only known from the type locality, off Wokam Island, Indonesia, in about 1788 m depth.

#### Sternaspis scutata

(Ranzani, 1817) emended

http://species-id.net/wiki/Sternaspis_scutata

Figure 13

**Figure 13.**
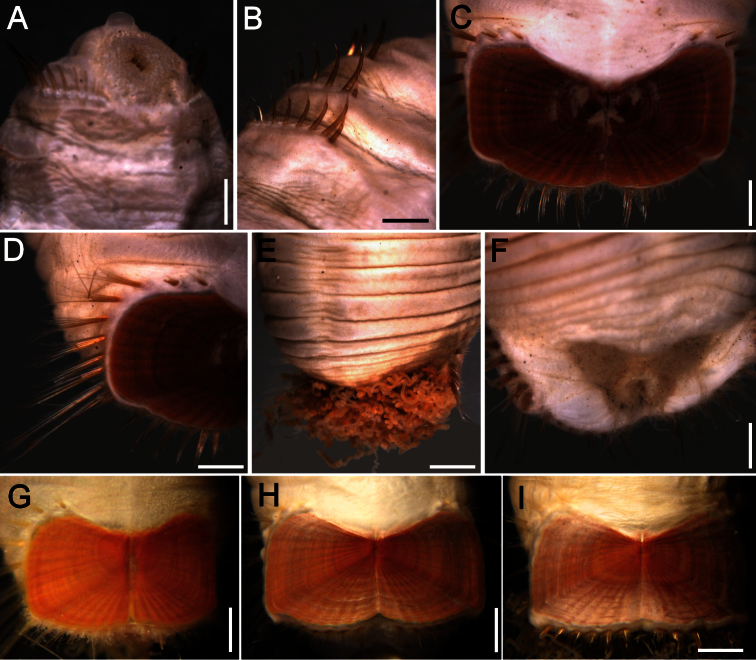
*Sternaspis scutata* (Ranzani, 1817), neotype (RBCM 005-140-001) **A** Anterior end, ventral view **B** Same, chaetae of first three chaetigers **C** Same, ventro-caudal shield **D** Paraneotype, ventro-caudal shield, oblique lateral view showing chaetal bundles **E** Neotype, posterior region, dorsal view **F** Another paraneotype, branchiae removed to show branchial plates **G–I** Non-type specimens (IRFA-STE 015), ventro-caudal shields. Bars: **A** 1.9 mm **B** 1.7 mm **C, D, F** 0.7 mm **E, I** 1.3 mm **G** 0.5 mm **H** 1.1 mm.

Thalassema scutatus Ranzani, 1817: 1458–1462, Pl. 11, figs 10–13.
Sternaspis scutata Claparède 1869: 95–96, Pl. 31, fig. 9; Rietsch 1882: 1–84, Pls. 18–23; Fauvel 1927: 216–218, fig. 76; 1934: 60; Townsend et al. 2006: 282–284, figs 1–2.

##### Type material.

**Eastern Mediterranean Sea, Aegean Sea**. Neotype (RBCM 005-140-001) and 9 paraneotypes (RBCM 005-140-002), Turkey, Izmar Bay, 38°30'00"N, 26°50'00"E, 33 m, 11-VII-2000.

**Additional material**. **Aegean Sea, Turkey.** 14 spec. (RBCM 005-139-001), Izmar Bay, 38°30'N, 26°50'E, 33 m, 11-VII-200. **Croatia.** 7 spec. (ECOSUR 2645), Rovigno d'Istria, VI-1983, J. Vidakovic & D. Zavodnik, coll. 2 spec. (ECOSUR 2646), off Rijeka, X-1981, P. Gillet, coll. 2 spec. (ECOSUR 2647), Rovigno d'Istria (no further data). **France**. 2 spec. (ZMA 1374), Bretagne. **Italy.** 8 spec. (MNHL 766), Gulf of Naples, 1888. Five spec. (ZMA 1373), Naples, 1893. 3 spec. (ZMA 1372), Triest. Five spec. (ZMA 1373), Bay of Naples, 1893. 2 spec. (ZMUC), Bay of Muggia, 1883. 1 spec. (ZMUC), Naples, Stazione Zoologica, 1882. 9 spec. (RBCM 006-008-001), Bay of Salerno, 40°29'N, 14°46'E, VIII-2002. 3 spec. (ANSP 1880), Bay of Naples. 9 spec. (RBCM 006-008-001), Bay of Salerno, 40°29'N, 14°46'E, VIII-2002. 4 spec. (IRFA-STE 015), Rijika, Oct. 1981. **Portugal.** 10 spec. (SMNH 50689), Lisboa, Tajo, 9-36 m, 1869.

##### Description.

Neotype (RBCM 005-140-001) with anterior region often swollen, bulbous compared to the remaining segments, with a constriction at septum between segments seven and eight. Body usually smooth, white, leathery, sometimes covered by minute cuticular papillae, especially behind seventh segment and near shield on dorsal side; posterior region slightly darker. Body papillae small, evenly spaced. Body up to 35 mm long, 18 mm wide, about 30 segments.

Prostomium hemispherical, without eyespots, opalescent, translucent (Fig. 13A). Peristomium rounded, flattening at the position of the mouth, devoid of papillae. Mouth circular, completely covered with minute papillae, extends from prostomium to edge of second segment.

First three chaetigers with over 10 bronze, widely separated, slightly falcate hooks, each with subdistal dark area (Fig. 13B), more evident in smaller specimens. Larger specimens with paler subdistal areas. Genital papillae protrude ventrally from body wall between segments 7 and 8. Pre-shield region with 7 segments, sometimes bearing a bundle of small, short, fine capillary chaetae laterally.

Ventro-caudal shield flat (Fig. 13C), ribbed, with concentric lines; suture restricted to anterior region. Anterior margins truncate, straight; anterior depression deep; anterior keels not exposed. Lateral margins straight, not expanded medially. Fan smooth, markedly projected beyond posterior corners, with margin smooth, barely crenulated (Fig. 13C, D).

Marginal shield chaetal fascicles include 10 lateral ones, chaetae in an oval arrangement, and six posterior fascicles, chaetae in a slightly curved arrangement. Chaetae of lateral fascicles hirsute, especially longer ones. Peg chaetae about as long as chaetae of first lateral chaetal fascicle and stout basally where chaetae emerge from cuticle, giving them a robust spine-like appearance. Additional chaetae delicate, in a small group.

Branchiae abundant; interbranchial papillae long, filamentous (Fig. 13E). Branchial plates diverging as half-fusiform areas (Fig. 13F).

##### Variation.

The ventro-caudal shield (Fig. 13G–H) has a fan with a median notch and its lateral parts extend beyond the posterior corners level, and this is a consistent pattern seen in all specimens regardless of size. The pigmentation is deep orange in smaller specimens (Fig. 13G) and becomes reddish in larger ones (Fig. 13H, I).

##### Neotype locality.

Izmar Bay, Aegean Sea, Turkey.

##### Remarks.

*Sternaspis scutata* (Ranzani, 1817) has been widely recorded and appears to be the most common species of *Sternaspis*. This is the oldest named species and researchers have suggested that *Sternaspis scutata* is a senior synonym of at least some of the other species of the family (Ushakov 1955; Hartman 1959), others have suggested that it is in fact the only species in the family (Pettibone 1954). These ideas are so widespread that over half of the worms loaned for this study were labelled as *Sternaspis scutata*. However, the species has not been redefined and in order to clarify the current confusion, a neotype is proposed, described and its diagnostic features are illustrated (ICZN 1999, Art. 75.3.1–75.3.3). Abbot Camilo Ranzani did not deposit the materials he described because it was not a current practice during those times (ICZN, Art. 75.3.4). However, Ranzani’s figure 13 clearly indicates that the ventro-caudal shield had a median, posterior notch, which is consistent with the proposed neotype (ICZN 1999, Art. 75.3.5), and distinct from the other Mediterranean species, *Sternaspis thalassemoides* Otto, 1821, because it has a rather straight posterior margin. This feature is consistent and has been found in the studied materials; they included specimens from the eastern Italian coast, which would be similar to the original type locality (Adriatic Sea). However, the best specimen was selected as neotype and it was collected in the Aegean Sea, some distance from the original type locality (ICZN 1999, Art. 75.3.6). As stated above, there were no differences among the materials studied. The neotype and additional paraneotypes have been deposited in the Royal British Columbia Museum (ICZN 1999, Art. 95.3.7).

As stated above, *Sternaspis scutata* differs from *Sternaspis thalassemoides* by shield features, especially regarding their fan development; in *Sternaspis scutata* it is notched and markedly expanded beyond the level of the posterior corners, whereas in *Sternaspis thalassemoides* it is truncate, entire, and not expanded beyond the posterior corners level. Further, *Sternaspis scutata* is unique in the genus by a combination of features of their shields: the anterior margins are truncate, the lateral margins are straight or barely rounded, and the posterior margin and fan are markedly expanded beyond the posterolateral corners.

##### Distribution.

Mediterranean Sea to the English Channel, 9–36 m depth. Deeper water records from the Eastern Mediterranean (Ben-Eliahu and Fiege 1995) deserve a careful comparison to define if they are conspecific with the shallow water material. Some records from non-Mediterranean or Northeastern Atlantic localities might belong to other, probably undescribed species. Thus the following records need to be checked: Arctic and Subarctic waters (Wesenberg-Lund 1950a: 104–105, 1950b: 46, 1951: 98, 1953: 88), Northwestern Pacific (Ushakov 1955: 353–354, fig. 131; Levenstein 1961: 167, 1966: 59, Buzhinskaja 1985: 166; Imajima 2005: 91), or Northeastern Pacific Ocean (Hartman 1971: 1422), Western Pacific (Gallardo 1968: 114), Red Sea (Fauvel 1957: 218), Indian Ocean (Wesenberg-Lund 1949: 345–346; Fauvel 1932: 213, 1953: 401–402, fig. 210a–g; Hartman 1976a: 199, 1976b: 627), Western Central (Gilbert 1984: 45.3–45.4, fig. 45.2a–f; Ibarzabal 1986: 14), Eastern Central (Fauvel 1936: 88), southeastern Atlantic (Day 1967: 648, fig. 31.1a–d), from New Zealand (Augener 1926: 283–286, fig. 22), and from the Antarctic Ocean (Hartman 1966: 55, Pl. 18, fig. 1; Hartman 1967: 141; Hartmann-Schröder 1986: 85; Hartmann-Schröder and Rosenfeldt 1989: 76, 1991: 77; Gambi and Mariani 1999: 238).

#### Sternaspis spinosa

Sluiter, 1882, emended

http://species-id.net/wiki/Sternaspis_spinosa

Figure 14

Sternaspis spinosa Sluiter, 1882: 277, Pl. 1, fig. 1.
Sternaspis scutata : Gallardo 1968: 114 (*partim*).

##### Type material.

**Indonesia**. Neotype (NHM 1889.6.15.52-36), Java, Bay of Batavia, “Batavia Roads”, outside Jakarta, 30 m, mud, 1889, purchased from Dr. Sluiter.

##### Additional material.

**Indonesia**. 1 spec. (ZMA 1491), Irian Jaya, Strait of Galewo, near Seget, 31 m, Stn 163. **Thailand**. 8 spec. (PMBC C1-0S), west of Takua Pa, 9°00'00"N, 98°02'00"E, 41 m, 17-IV-1998. 1 spec. (PMBC B2-0S), Andaman Sea, NW off Takua Pa, 9°14'00"N, 98°00'00"E, 45 m, 17-II-1998. 1 spec. (PMBC C2-0S), Andaman Sea, W off Takua Pa, 9°00'00"N, 97°56'00"E, 60 m, 17-II-1998. **Vietnam.** 1 spec. (LACM n 11878), Sta. 126 (no coord.), 17 m, mud, 11-II-1960. 1 spec. (LACM n 11884), Sta. 173 (no coord.), 32 m, sand, 25-II-1960. **Australia.** 1 spec. (AM W 202648), Queensland, Shoalwater Bay, Triangular Islets. One spec. (AM W 28515), Queensland, Coral Sea, Capricorn Channel, southeast of Swains Reef, 22°31'07"S, 152°42'38"E, 78 m. 2 spec. (AM W 28516), Queensland, Coral Sea, Capricorn Channel, SE off Swains Reef, 22°03'27"S, 152°33'54"E, 100 m. 1 spec. (AM W 28512), Queensland, Coral Sea, Capricorn Channel, 6.8 miles NW off Pine Peak Island, 21°27'30"S, 15°00'48"E, 42 m. 1 spec. (AM W 28517), Queensland, Juno Bay, near Ingham, 18°41'00"S, 146°30'00"E. 1 spec. (AM W 28509), Western Australia, 72 nautical miles NW off Dampier, 19°28'54"S, 116°29'24"E, 110 m.

##### Description.

**Figure 14.**
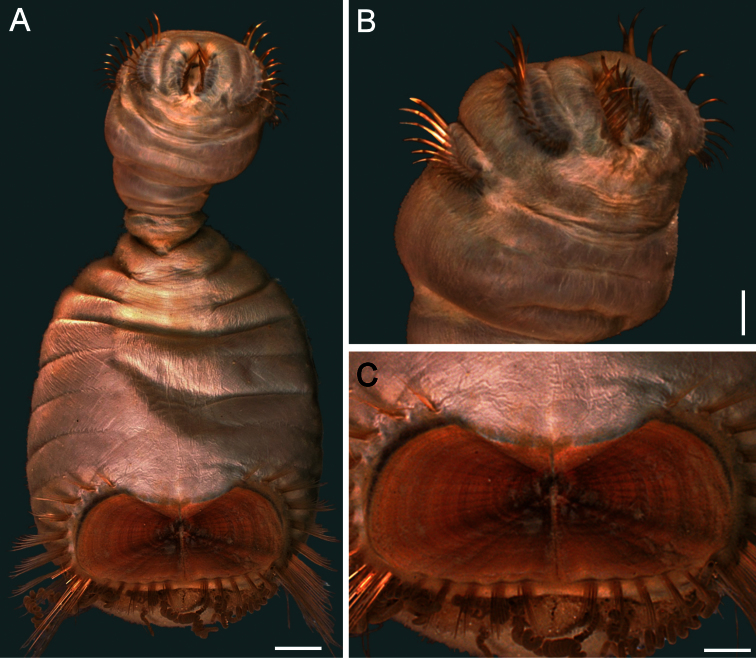
*Sternaspis spinosa* Sluiter, 1882, Neotype (NHM 1889.6.15.52, No. 36) **A** Complete, ventral view **B** Same, anterior end, exposed, oblique lateral view **C** Same, ventro-caudal shield. Bars: **A** 1.4 mm **B** 1.0 mm **C** 0.8 mm.

Neotype (NHM 1889.6.15.52, No. 36) without adhering sediment and bright white or cream in colour (Fig. 14A), larger specimens sometimes darker. Anterior segments without cuticular papillae, some present on segments 6–8, short, evenly spaced. Following segments with well-defined single rows of clustered, longer filamentous, white papillae; larger specimens with median segments papillae eroded. Neotype 17.5 mm long, 8.7 mm wide; body up to 20 mm long, 8.5 mm wide, about 29 segments.

Prostomium hemispherical, opalescent in larger individuals, translucent in smaller individuals. Peristomium rounded, small. Mouth oval, covered by papillae (bright white in smaller specimens), extends from prostomium to anterior edge of second segment.

First three anterior chaetigers with over 10 bronze, widely separated, falcate hooks (paler in smaller specimens), each with subdistal dark areas (Fig. 14B). Genital papillae protrude ventrally from intersegmental furrow between segments 7 and 8. Pre-shield region with 7 segments, with short delicate fascicles of a few capillary chaetae on some specimens.

Ventro-caudal shield pale brown, usually clean, sometimes with adhered sediment; ribs not well-defined, concentric lines present; suture extended throughout shield, barely visible. Anterior margins angular; anterior depression shallow; anterior keels exposed (Fig. 14C). Lateral margins rounded, expanded posteriorly. Fan truncate, barely projected beyond posterior corners, margin crenulated.

Marginal shield chaetal fascicles include 10 lateral ones, chaetae in a slightly curved arrangement, and five posterior fascicles, chaetae in a narrow oval arrangement. Peg chaetae narrow, sometimes as long as posterior shield chaetae. Additional delicate capillary chaetae between peg chaetae and first posterior fascicle of shield chaetae.

Branchiae tightly coiled, protrude from two very narrow, widely divergent plates on either side of anus. Interbranchial papillae abundant, on either side of anus.

##### Neotype locality.

Bay of Batavia, Java, Indonesia.

##### Remarks.

*Sternaspis spinosa* Sluiter, 1882 has been in doubt ever since the original description because it was described and illustrated with long palp-like appendages; however, this type of appendage has not been reported for any other species, many authors doubt their presence and, by extension, even of the species delineation itself. However, the analysis of the available material has led us conclude that the species is distinct and in order to clarify its taxonomic status (ICZN 1999, Art. 75.3.1) a neotype has been selected, described and its diagnostic features have been illustrated (ICZN 1999, Art. 75.3.2–75.3.3). There is no type material available, as indicated by Petersen (2000: 321), but Sluiter identified some other specimens and we have selected one of them as the neotype (ICZN 1999, Art. 75.3.4). This specimen and all others from the same lot resemble each other and conform to the original materials, at least regarding the shape of the ventro-caudal shield. Further, because Sluiter identified some of them, we are confident they agree with the original (and now lost) materials (ICZN 1999, Art. 75.3.5). The proposed neotype was collected in the same locality, Bay of Batavia, Java, as the original materials (ICZN 1999, Art. 75.3.6), and it was deposited in the Natural History Museum, London (ICZN 1999, Art. 75.3.7).

There are many features separating *Sternaspis spinosa* from other species, such as the flatter, less ribbed shield, with 10 lateral and five posterior fascicles of shield chaetae, well-defined rows of papillae and longer peg chaetae. The characteristics of *Sternaspis spinosa* are distinctive compared to *Sternaspis costata*, and we regard them as separate species. Concerning the presence of palps, Fauvel (1927) did not consider *Sternaspis spinosa* to be in the family, and Petersen (2000) suggested that Sluiter may have examined a damaged specimen where a portion of the digestive tract had been extruded to the exterior. However, according to Rouse and Pleijel (2001), these appendages may not be part of the gut. There is a thick cuticle, musculature and blood supply to the appendages, which would indicate that they are moveable and have a function in digging or anchoring the body in the sediment. There is no groove along the appendages, but the area where they attach near the mouth is heavily ciliated. Sluiter comments that only one of the specimens he collected had these appendages, and that they may have been lost in others due to the method of collection. Petersen (2000) indicated that there are three dried out specimens with Sluiter’s material at the Zoological Museum, University of Amsterdam, but none have the appendages or any trace of them. Sluiter also included two very robust peg chaetae protruding from the underside of the shield near the posterolateral margins. It is unfortunate the types were not located because this species has not been collected or reported since. However, no evidence of the palps, including scars or traces were observed on other specimens (NHM 1889.6.15.52, No. 36)) identified by Sluiter as *Sternaspis spinosa*.

On the other hand, *Sternaspis spinosa* resembles *Sternaspis africana* by having shields with deep anterior depressions and markedly expanded lateral shield margins. However, in *Sternaspis spinosa* the shield integument is transparent and both ribs and concentric lines are visible, whereas in *Sternaspis africana* the ribs are barely noticeable. Further, the shield of *Sternaspis spinosa* has a posterior margin straight, at same level as margin of shield resembling *Sternaspis princeps*, *Sternaspis rietschi*, *Sternaspis spinosa*, *Sternaspis thalassemoides* and *Sternaspis thorsoni* sp. n. However, *Sternaspis spinosa* can be distinguished from them as its shield is much wider than long and by having its anterior keels exposed.

##### Distribution.

Queensland Australia, Coral Sea, Thailand in the Andaman Sea, Vietnam and Indonesia, 17–110 m depth.

#### Sternaspis thorsoni sp. n.

urn:lsid:zoobank.org:act:F1AB89B5-56F7-47F5-B3C2-D9F396CCAD39

http://species-id.net/wiki/Sternaspis_thorsoni

Figure 15

Sternaspis scutata : Wesenberg-Lund 1949: 345–346 (*non*Ranzani 1817), Fauvel 1932: 213 (*non*Ranzani 1817, *partim*).

##### Type material.

**Arabian (Iranian or Persian) Gulf.** Holotype (ZMUC 2221), 55.6 km NNW of buoy near Jask, Iran, Sta. 76 (25°45'N, 57°12'E), 110 m, loose, brown clay, 21-IV-1937, G. Thorson, coll. 6 paratypes: 1 (ZMUC 2222), juvenile, 4 km S Bushire outer Light-buoy, Sta. 28 (no coord.), 7 m, 18-III-1937, G. Thorson, coll. 1 (ZMUC 2223), juvenile, Henjom Island, Strait of Hormuz, Sta. 59 (26°36'N, 55°42'E), 31 m, 10-IV-1937, G. Thorson, coll. 1 (ZMUC 2224), adult, Patrick Steward Bank, Sta. 71B (26°41'N, 56°16'E), 69 m, gray mud, 19-IV-1937, G. Thorson, coll. 3 spec. (ZMUC 2225), juveniles, 17 km SSE off mountain Kuh-i-Namak Sar range, Sta. 114 (27°00'30N, 56°03'E), 13 m, sand with little clay, 4-IV-1938, G. Thorson, coll.

**Additional material. Arabian (Iranian or Persian) Gulf**. 1 spec. (ZMUC), juvenile, 3 km SSW off Kharg, Sta. 8 (29°14'N, 50°19'E), 40 m, soft, grey clay, 5-III-1937, G. Thorson, coll. 8 spec. (ZMUC), juveniles, partly dehydrated, 5.5 km SE Bushire outer Light-buoy, Sta. 28 (no coord.), 7 m, grey-brown clay, 18-III-1937, G. Thorson, coll. 3 spec. (ZMUC), juveniles, off road to Bender Abbas, Sta. 64B (no coord.), 3 m, soft clay, 16-IV-1937, G. Thorson, coll. 3 spec. (ZMUC), juveniles, off road to Bender Abbas, Sta. 64Bx, 3 m, soft clay, 16-IV-1937. 2 spec. (ZMUC), 11 km ENE from Quishim Light-buoy, Sta. 65 (27°01'N, 56°00'E), 18 m, dark sand with clay, 16-IV-1937, G. Thorson, coll. Andaman Sea. 5 spec. (MNHN 454), Andaman Islands, no further data.

##### Description.

**Figure 15.**
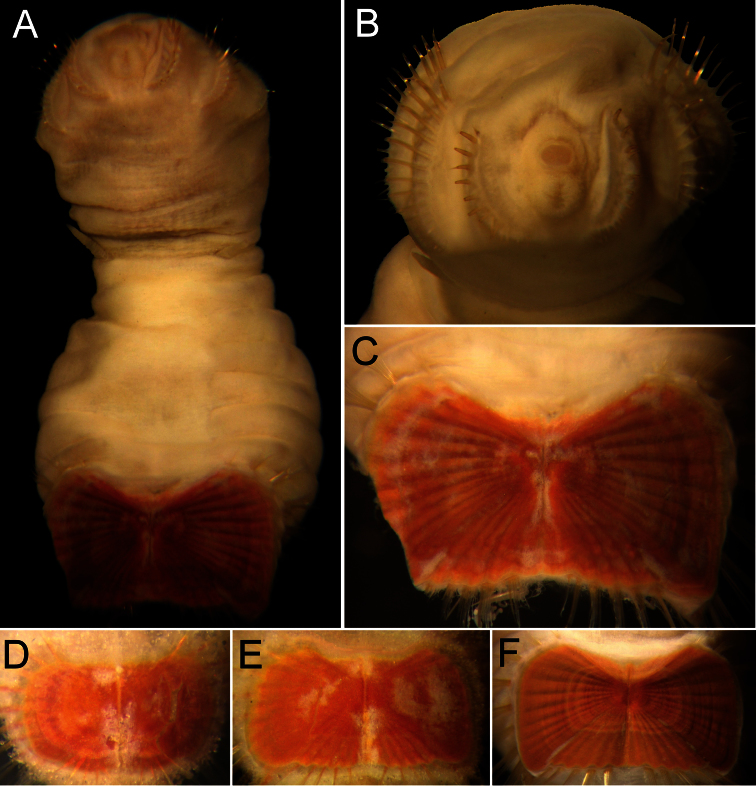
*Sternaspis thorsoni* sp. n. **A** Holotype (ZMUC 2221), ventral view **B** Same, anterior end, frontal view **C** Same, ventro-caudal shield **D** Mon type specimen (ZMUC Sta. 64), ventro-caudal shield **E** Paratype (ZMUC 2224), ventro-caudal shield **F** Another paratype (ZMUC 2223), ventro-caudal shield. Bars: **A**, **F** 0.8 mm **B** 0.6 mm **C** 0.5 mm **D** 0.3 mm **E** 0.4 mm.

Holotype with body whitish or grayish (Fig. 15A), introvert slightly darker, integument granulose; abdomen smooth. Papillae minute, abundant, short and longer, filiform, uniformly distributed especially on abdomen. Body 14 mm long, 5 mm wide, with about 30 segments.

Prostomium small, without eyespots. Peristomium rounded, depressed below mouth, without papillae (Fig. 15B). Mouth circular, completely covered with minute papillae, continued ventrally forming an arc.

First three chaetigers with 16–20 hooks, thin, pale with a subdistal barely darker band (Fig. 15B). Genital papillae protrude ventrally from intersegmental groove between segments 7 and 8. Pre-shield region with 7 segments, without fascicles of fine capillary chaetae.

Ventro-caudal shield previously sliced along posterior right corner, with radiating oblique ribs and concentric lines; suture restricted to anterior region (Fig. 15A, C). Anterior margins angular; anterior depression deep; anterior keels visible, but not exposed. Lateral margins slightly expanding posteriorly. Fan truncate, not extending beyond posterior corners, crenulated, not projected outwardly; median notch shallow or indistinct.

Marginal chaetal fascicles include 10 lateral ones, chaetae ovally arranged, and seven posterior fascicles, chaetae in a slightly curved arrangement. First two lateral fascicles emerge from ventral edge of shield. Lateral fascicle with long hirsute chaetae. Peg chaetae in posterior corner region.

Branchiae mostly removed, spirally arranged.

##### Type locality.

Off Jask, Iran.

##### Variation.

Smaller paratypes have better defined body papillae which are larger, especially on abdominal segments. Likewise, paratypes exhibit ventro-caudal shields which are rounded without surface features in smaller specimens (Fig. 15D), with a suture well defined but little definition of anterior margins and reduced development of posterior corners. Larger specimens show better definition of anterior margins and more developed posterior corners, together with crenulations of the fan margin, but concentric lines are not well-defined (Fig. 15E). Larger specimens have all surface ornamentation features, together with well defined acute anterior margins and posterior corners extended beyond the fan level, and more definite resolution of fan crenulations (Fig. 15F) than smaller specimens.

##### Etymology.

The species name is derived after the late Dr. Gunnar Thorson in recognition of his important contributions to benthic ecology, especially with regards to reproduction and larval development (Thorson 1946, 1950), and comparative studies of benthic communities where he coined the concept of parallel communities (Thorson 1957). He also made many collecting trips in temperate and tropical communities and the specimens used for this description were based on his collections. The epithet is a noun in the genitive case.

##### Remarks.

The shield of *Sternaspis thorsoni* sp. n.has a truncate posterior margin resembling *Sternaspis princeps*, *Sternaspis rietschi*, *Sternaspis spinosa* and *S thalassemoides*. As indicated above, *Sternaspis spinosa* is characterised by having a shield markedly wider than long and by having exposed its anterior keels. Further, *Sternaspis thorsoni* is unique as it has more abundant, pale delicate introvert hooks, whereas the other species have fewer, thicker, darker hooks.

Fauvel (1932: 213) indicated three shield colour variants. The only specimens available, collected in the Andaman Islands, are all conspecific and almost completely fit this new species description, although the larger specimen has a marked notch on the shield’s fan.

##### Distribution.

Arabian Sea, in muddy bottoms in shallow water (3–110 m). Probably reaching as far as the Andaman Sea.

#### Genus Caulleryaspis gen. n.

urn:lsid:zoobank.org:act:0C0920FC-3CFE-465F-9D8D-B5640CC00E0E

http://species-id.net/wiki/Caulleryaspis

##### Type species.

*Caulleryaspis gudmundssoni* sp. n.

##### Diagnosis.

Sternaspids with introvert hooks tapered. Pre-shield region with 7 segments. Ventro-caudal shield flexible with abundant sediment particles firmly adhered.

##### Remarks.

*Caulleryaspis* gen. n. differs from *Sternaspis* and *Petersenaspis* gen. n. because the shield is soft and has abundant sediment particles firmly adhered to it, whereas the two other genera have shields stiff, without sediment particles firmly adhered to it. Other differences were indicated in the key above.

*Caulleryaspis* includes, besides the type species, *Caulleryaspis gudmundssoni* sp. n. from Iceland, *Caulleryaspis laevis* (Caullery, 1944) comb. n. from Indonesia. These species can be separated because of differences in the shield shape (see key below).

##### Etymology.

The genus name is to honor Dr Maurice Caullery, in recognition of his studies on polychaete reproductive biology and taxonomy, and especially because of his monograph on the *Siboga* Expedition, which took him 40 yr, and contained many new species. *Caulleryaspis* is a free combination of his last name and the second part of *Sternaspis* (Gr. shield) to stress the affinity with the stem genus. Gender: feminine.

##### Key to species of *Caulleryaspis* gen. n.

(distribution in parenthesis)

**Table:** 

1	Shield with anterior depression deep; peg chaetae markedly robust	*Caulleryaspis gudmundssoni* sp. n. (North Atlantic Ocean, Iceland)
–	Shield with anterior depression shallow; peg chaetae indistinct	*Caulleryaspis laevis* (Caullery, 1944) comb. n. (Indonesia)

#### Caulleryaspis gudmundssoni sp. n.

urn:lsid:zoobank.org:act:F93976AE-B573-4ACE-8F8A-6BB6149CD71E

http://species-id.net/wiki/Caulleryaspis_gudmundssoni

Figure 16

##### Type material.

**Atlantic Ocean, Iceland**. Holotype (IMNH 10280), and 5 paratypes (IMNH 10282), BIOICE Program, R.V. Bjarni Saemundsson, Sta. 2429 (63°02.30'N, 21°50.80'W), 1072 m, sandy silt, 3-VII-1993. 16 additional paratypes as follows: 1 (IMNH 10281), BIOICE Program, R.V. Bjarni Saemundsson, Sta. 2430 (63°07.90'N, 19°57.20'W), 1016 m, no sediment data, 3-VII-1993. 3 (IMNH 10283), BIOICE Program, R.V. Bjarni Saemundsson, Sta. 2404 (63°02.30'N, 21°50.80'W), 827 m, sandy silt, 1-VII-1993. 2 (IMNH 10284), BIOICE Program, R.V. Bjarni Saemundsson, Sta. 2415 (63°00.18'N, 21°54.63'W), 819 m, no sediment data, 2-VII-1993. 1 (IMNH 10285), BIOICE Program, R.V. Bjarni Saemundsson, Sta. 2414 (63°00.30'N, 21°00.76'W), 808 m, sandy silt, 2-VII-1993. 4 (2 IMNH 10286, 2 MNHN 1555), BIOICE Program, R.V. Bjarni Saemundsson, Sta. 2475 (63°04.20'N, 21°34.90'W), 842 m, sandy silt, 5-VII-1993. 1 (IMNH 10287), BIOICE Program, R.V. Bjarni Saemundsson, Sta. 2409 (62°52.37'N, 21°43.62'W), 1080 m, silt with large rock, 2-VII-1993. 1 (IMNH 10288), BIOICE Program, R.V. Bjarni Saemundsson, Sta. 2468 (63°10.00'N, 21°30.90'W), 452 m, sandy silt, 5-VII-1993. 3 (IMNH 10289), BIOICE Program, R.V. Bjarni Saemundsson, Sta. 2431 (63°04.08'N, 19°51.33'W), 1207 m, sandy silt, 3-VII-1993.

##### Description.

**Figure 16.**
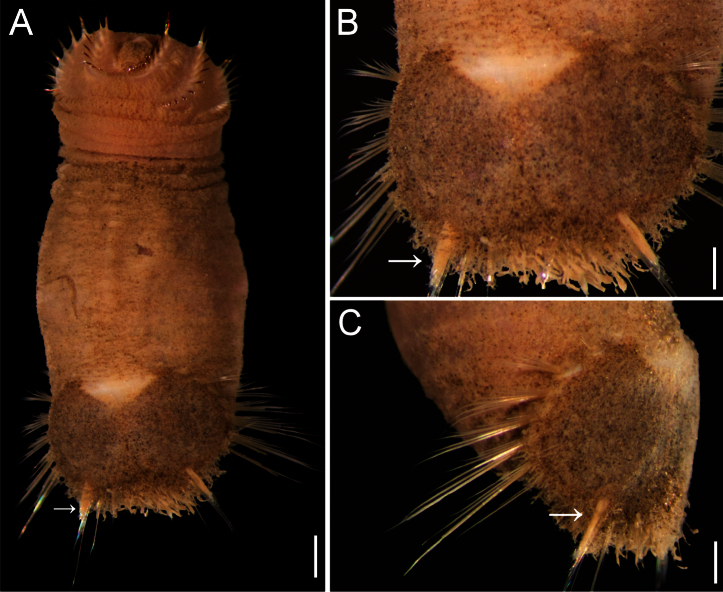
*Caulleryaspis gudmundssoni* sp. n., holotype (IMNH 10280) **A** Ventral view (arrow points peg chaetae) **B** Same, ventro-caudal shield (arrow points peg chaetae) **C** Same, posterior region, lateral view (arrow points peg chaetae). Bars: **A** 1.4 mm, **B** 0.8 mm **C** 0.9 mm (Photos: Gudmundur Vidir Helgason).

Holotype (IMNH 10280) with body stout and of equal width over the anterior, preshield and shield regions (Fig. 16A). Colour tan, speckeld with small sediment particles. Abundant, minute cuticular papillae, incorporating fine sediment particles, except in the areas where introvert hooks emerge. Segments seven and eight with more cuticular papillae near genital papillae. Cuticular papillae not present, even dorsally near ventro-caudal shield, but a few may be present on more posterior segments. Body 7.5 mm long, 3.5 mm wide, about 28 segments.

Prostomium hemispherical, conspicuously extended, white, opaque. Peristomium small, oval, bearing some papillae closer to mouth. Mouth oval, small, completely covered by papillae, extends from prostomium to anterior border of second segment.

First three chaetigers with 10–15 falcate, flat introvert hooks per bundle, closely associated, each with subdistal dark areas. Genital papillae protrude ventrally from intersegmental furrow between segments 7 and 8. Pre-shield regions with 7 segments, smooth, some bearing small groups of fine, short capillary chaetae.

Ventro-caudal shield completely covered by a thick coating of adhered particles, unusually flexible; suture not visible (Fig. 16A–C). Anterior margins apparently rounded (shape blocked by sediment cover); anterior depression deep; anterior keels not exposed. Ribs, concentric lines or fan not visible. Lateral margins rounded, expanded medially, reduced posteriorly. Fan truncate, barely reaching posterior corners. Other features not visible.

Marginal chaetal fascicles include 10 lateral ones, and only three short, small posterior fascicles (other ones apparently broken), each with 3–4 chaetae concentrated near posterolateral edge of shield. Peg chaetae robust, stout in cross basal section, pale gold, emerge directly from a raised portion of shield, close to posterior margins (Fig. 16A–C). Additional two couplets or triplets of fine short capillary chaetae between peg chaetae and first posterior shield chaetae fascicles.

Branchiae few, very slender coiled filaments on two roughly parallel plates; longer, more slender, straight filamentous papillae closer to anus.

##### Etymology.

The species name is derived after Dr. Gudmundur Gudmundsson, from the Iceland Natural History Museum in recognition of his long-standing support for our research activities. The epithet is a noun in the genitive case.

##### Type locality.

Off southeast of Vestmannaey jar, Iceland, 1072 m.

##### Remarks.

*Caulleryaspis gudmundssoni* sp. n. resembles *Caulleryaspis laevis* (Caullery, 1944) comb. n. because both species have sediment particles covering their soft shields. These species differ in the relative development of the anterior shield depression and especially on the relative development of peg chaetae. In *Petersenaspis gudmundssoni* the anterior depression is deep and the peg chaetae are robust, being easily noticed over the shield itself, whereas in *Caulleryaspis laevis* the anterior depression is shallow and the peg chaetae are not well developed.

##### Distribution.

Only known from the type locality off southwest Iceland, in sediments of 452–1207 m depth.

#### Caulleryaspis laevis

(Caullery, 1944) comb. n.

http://species-id.net/wiki/Caulleryaspis_laevis

Figure 17

Sternaspis laevis Caullery, 1944: 67–68, fig. 52; Bleeker and van der Spoel 1992: 159.

##### Type material.

**Indonesia**. Lectotype of *Sternaspis laevis* (ZMA 1535), and one paralectotype (ZMA 5530), Sumbawa Island, Bay of Bima (08°27.5'S, 118°43.5'E), 55 m, Sta. 47. One paralectotype (ZMA 1491), Irian Jaya, Strait of Galewo, near Seget (01°24'S, 130°58'E), 31 m, R. V. Siboga, Sta. 163 (dried-out).

##### Additional material.

**Thailand, Andaman Sea**. 2 spec. (ZMUC), Stn. J47-0S, 7°15'N, 98°51'E, 61 m, 4-V-1996. 1 spec. (ZMUC), Stn. L57-0S, 6°44'N, 99°05'E, 56 m, 5-V-1996. 5 spec. (PMBC J1-OS), S off Phuket, 7°15'00"N, 99°04'00"E, 39 m, 23-II-1998. 2 spec. (PMBC 12-AT), S off Phuket, 7°30'00"N, 98°29'00"E, 62 m, 26-II-2000. 5 spec. (PMBC K1-0S), W off Kantang, 7°00'00"N, 99°16'00"E, 41 m, 24-II-1998. 2 spec. (PMBC GI-OS), W off Thalang, 7°59'00"N, 98°12 ‘00"E, 46 m, 20-II-1998. 2 spec. (PMBC K1-HS), SW off Kantang, 7°00'00"N, 99°16'00"E, 43 m, 27-II-2000. 2 spec. (PMBC C1-OS), W off Takua Pa, 9°00'00"N, 98°02'00"E, 41 m, 17-II-1998. 5 spec. (PMBC B2-0S), NW off Takua Pa, 9°14'00"N, 98°00'00"E, 45 m, 17-II-1998. 4 spec. (PMBC C2-0S), W off Takua Pa, 9°00'00"N, 97°56'00"E, 60 m, 17-II-1998. 2 spec. (ZMUC J47-0S), SW off Kantang, 7°15'00"N, 98°51'00"E, 62 m, 04-V-1 996. 1 spec. (ZMUC L57-0S), SW off Kantang, 6°44'00"N, 99°05'00"E, 56 m. **Australia, Queensland**. Calliope R., N off Gladstone, 23°51'00"S, 151°10'00"E.1 spec. (AM W 8516), 26-VI-1975. 20 spec. (AM W 199324), 1974. 6 spec. (AM W 28511), 1974. 1 spec. (AM W 10295). 2 spec. (AM W l0296), Gladstone, Auckland Ck., 23°51'00"S, 151°14'00"E. 25 spec. (AM W 202648), Shoal water, Triangular Islets. **Coral Sea.** 1 spec. (AM W 28507), NE off Cairns, 16°36'00"S, 146°40'00"E, 147 m.

##### Description.

**Figure 17.**
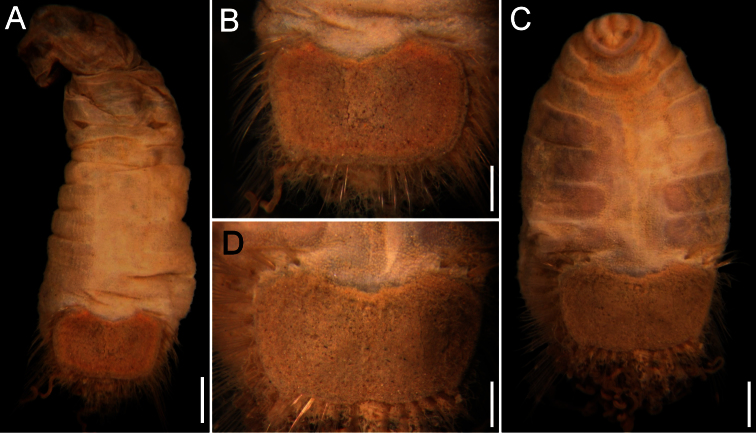
*Caulleryaspis laevis* (Caullery, 1944) comb. n. **A** Lectotype (ZMA 1535), ventral view **B** Same, ventro-caudal shield **C** Paralectotype (ZMA 5530), ventral view **D** Same, ventro-caudal shield. Bars: **A** 1 mm **B** 0.5 mm **C** 1.1 mm **D** 0.6 mm.

Lectotype (ZMA 1535), with anterior end exposed, damaged; first five anterior segments light grey, opalescent with few cuticular papillae (Fig. 17A). Starting with segment seven, remainder of body darker grey or tan, and leathery in appearance. Cuticle covered with minute filamentous cuticular papillae over most of surface, especially on segments seven and eight, where papillae become longer. Two rows of loosely arranged dark spots with filamentous cuticular papillae on posterior segments starting with segment eight (better developed in paralectotype ZMA 1491). On segments dorsal to ventro-caudal shield, spots consist of slightly longer cuticular papillae with encrusting sediment at bases. Body up to 12.5 (6.5) mm long, 5.5 (2) mm wide, 29 segments.

Prostomium hemispherical, opalescent, light grey in colour. Peristomium rounded, raised at position of mouth, with a few papillae around base of prostomium. Mouth papillated, circular and small, positioned halfway between prostomium and anterior edge of segment two.

First three chaetigers with about six to ten larger, and five or more smaller, bronze, widely separated, slightly falcate introvert hooks per bundle, most with tips broken, with subdistal darker areas. Genital papillae protrude ventrally from intersegmental groove between segments 7 and 8 (Fig. 17A, C). Pre-shield region with 7 segments, without rows of fine capillary chaetae.

Ventro-caudal shield covered by fine papillae, with sediment particles firmly adhered on it; anterior margins rounded; anterior depression shallow or very shallow; suture not visible (Fig. 17B, D). Lateral margins rounded, medially expanded, narrowing posteriorly. Fan truncate, slightly expanded medially, margin smooth, with a shallow median notch (paralectotype ZMA 1491 with rust red in central area, with a wide bluish band of rings next, followed by another ring of rust red at outer margins, concentric lines not seen, basal layer porous).

Marginal chaetal fascicles include ten lateral ones, chaetae in a narrow oval arrangement, and five posterior fascicles, with chaetae in an offset linear arrangement, but roughly parallel to each other. Peg chaetae long, with a narrow base in cross section, emerge from cuticle, almost equalized to margin of shield. Additional delicate chaetae between peg chaetae and first bundle of posterior chaetae, almost included with peg chaetae.

Branchiae numerous, coiled, protrude from two widely separated plates, on dorsal surface adjacent to the ventro-caudal shield.

##### Remarks.

The original syntype series of *Sternaspis laevis* Caullery, 1944 contains two different species based on their ventro-caudal shields: three syntypes have an hirsute integument with abundant sediment particles firmly attached, and the shield basal layer is soft, porous, and another one has a shield with a stiff basal layer. In order to redefine the species delineation because these two shield patterns differ a lectotype has been selected (ICZN 1999, Art. 74.1), the term has been introduced in the materials section and in the description (ICZN 1999, Art. 74.7.1), described and illustrated (ICZN 1999, Art. 74.7.2) and the two other specimens are regarded as paralectotypes (ICZN 1999, Recomm. 74F). This proposal has been made to restrict the use of this species name to those specimens having hirsute shields with abundant, firmly attached sediment particles (ICZN 1999, Art. 74.7.3). The selected specimen (lectotype) corresponds to the originally illustrated specimen (ICZN 1999, Recomm. 74B).

Another syntype of *Sternaspis laevis* (ZMA 1491) is damaged, most body papillae were eroded, most shield fascicles chaetae were broken, its introvert is invaginated, and its papillae are arranged in transverse groups; the shield has a stiff layer, with concentric lines and ribs, showing a banded pigmentation. It resembles *Sternaspis spinosa* and does not belong to *Petersenaspis laevis*. On the other hand, of the ten syntypes of *Sternaspis laevis* var. *minor*, five (ZMA 1528), are very small specimens perhaps of *Caulleryaspis laevis*, but their small size complicates their positive identification; the other five syntypes (ZMA 1504), are dried-out, and their identification is even more problematic. Consequently, *Sternaspis laevis* var. *minor* must be regarded as indeterminable.

*Caulleryaspis laevis* (Caullery, 1944) comb. n. differs in two main characters from *Caulleryaspis gudmundssoni* sp. n.: the relative development of the anterior shield depression and the relative development of peg chaetae. In *Petersenaspis laevis* the anterior depression is shallow and peg chaetae are not well developed, making them difficult to be detected, whereas in *Petersenaspis gudmundssoni* the anterior depression is deep and peg chaetae are very robust, being easily noticed from the surrounding shield surface.

##### Distribution.

Andaman Sea to Southeastern Australia, 39–147 m depth. Kastoro et al. (1989) think this is a very common estuarine species in East Java, in 0.3–20.0 m, and salinities of 29.3–34.0 ‰.

#### Genus Petersenaspis gen. n.

urn:lsid:zoobank.org:act:7AE1C3E5-B68D-457A-AD75-08A956F5B736

http://species-id.net/wiki/Petersenaspis

##### Type species.

*Sternaspis capillata* Nonato, 1966.

##### Diagnosis.

Sternaspids with introvert hooks subdistally expanded. Pre-shield region with 8 segments. Ventro-caudal shield stiff with feebly developed ribs, and no concentric lines.

##### Remarks.

*Petersenaspis* gen. n. and *Sternaspis* have stiff shields, whereas *Caulleryaspis* has soft shields. However, *Petersenaspis* differs from *Sternaspis* because its introvert hooks are subistally expanded, there are 8 segments in the pre-shield region, and the shield has deeply developed ribs but no concentric lines, whereas in *Sternaspis* introvert hooks are tapered, there are 7 segments in the pre-shield region, and the shield has well developed ribs, often with concentric lines.

As stated above, *Petersenaspis* gen. n. includes, besides the type species, *Petersenaspis capillata* (Nonato, 1966) comb. n., from Central and Southern Brazil, *Petersenaspis palpallatoci* sp. n. from the Philippine Islands, and another species which is characterized below. These species can be separated by the key below.

##### Etymology.

The genus name is to honor Dr Mary E. Petersen, in recognition of her many studies of polychaetes, including a valuable synthesis of *Sternaspis* species, and especially because of her long-term support of all our research activities. *Petersenaspis* is a free combination of her last name and the second part of *Sternaspis* (Gr. shield) to stress the affinity with the stem genus. Gender: feminine.

##### Key to species of *Petersenaspis* gen. n.

(distribution in parenthesis)

**Table:** 

1	Shield with anterior margin truncate; fan with median notch	*Petersenaspis capillata* (Nonato, 1966) comb. n. (Southwestern Atlantic Ocean, Brazil)
–	Shield with anterior margin projected forward; fan with median and lateral notches	*Petersenaspis palpallatoci* sp. n. (Philippine Islands)

#### Petersenaspis capillata

(Nonato, 1966) comb. n.

http://species-id.net/wiki/Petersenaspis_capillata

Figure 18

Sternaspis capillata Nonato, 1966: 79–83, figs l–9; Nonato and Luna 1970: 94, figs 87–88.

##### Type material.

**Brazil**. Two syntypes (MCEM 1333), Vitoria Island, 23°45'18"S, 44°00'54"W, 52 m, 1965.

##### Additional material.

**Brazil.** 1 spec. (MCEM 1309), Florianopolis, 27°45'51"S, 48°03'00"W, 95 m, 15-III-1998. 1 spec. (MCEM 1310), Ararangua, 29°15'00"S, 48°41'00"W, 101 m, 23-III-1998. 1 spec. (MCEM 1311), Florianopolis, 27°46'49"S, 47°40'45"W, 138 m, 16-III-1998. 1 spec. (MCEM 1312), Cricifuna, 28°41'22"S, 48°18'24"W, 109 m, 22-III-1998. 4 spec. (MCEM 1313), Imbituba, 28°05'00"S, 48°06'00"W, 100 m, 16-III-1998. 6 spec. (MCEM 1314), Imbituba, 28°05'00"S, 48°06'00"W, 100 m, 16-III-1998.

##### Description.

**Figure 18.**
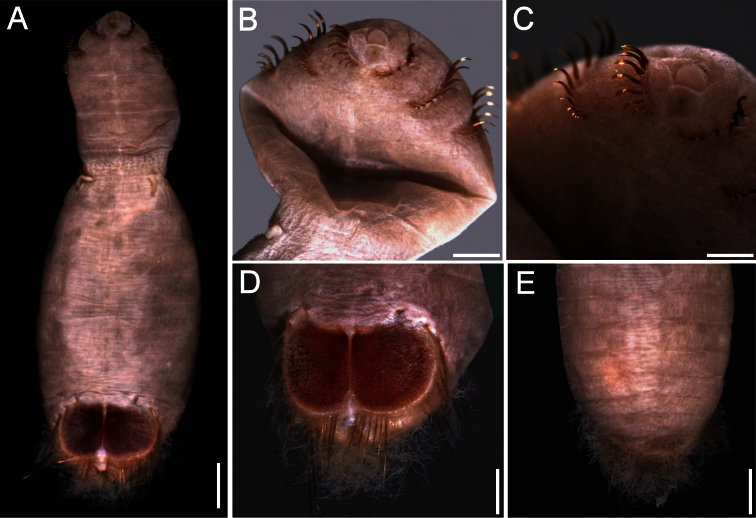
*Petersenaspis capillata* (Nonato, 1966) comb. n., syntypes (MCEM 1333) **A** Complete, ventral view **B** Another syntype, anterior end, ventral view **C** Same, enlargement to show subdistally expanded chaetae **D** Same, ventro-caudal shield, frontal view **E** Another syntype, posterior region, dorsal view. Bars: **A** 1 mm **B, D** 0.6 mm **C** 0.4 mm **E** 0.9 mm.

Syntypes (MCEM 1333) with body bright white, clean with barely visible minute filamentous papillae covering most of cuticle (Fig. 18A), more densely on segments 7 and 8. Faint single rows of clusters of papillae along dorsal surface of last few segments. Body up to 20 mm long, 4.5 mm wide, 33 segments.

Prostomium hemispherical, opalescent, conspicuous (Fig. 18B, C). Peristomium rounded, equalized at position of mouth, with some papillae. Mouth circular, extends from base of prostomium to anterior edge of first chaetiger.

First three chaetigers with about 10 bright bronze, recurved, spatulate hooks, without subdistal dark areas (Fig. 18B, C). Genital papillae protrude ventrally from body wall between segments 7 and 8. Pre-shield region with 8 segments, with single lateral bundles of few capillary chaetae protruding from body wall.

Ventro-caudal shield brick red, papillose, with ribs faintly defined but no concentric lines, nor sediment particles; suture extends throughout shield. Anterior margins rounded; anterior depression very shallow; anterior keels not exposed. Lateral margins rounded, expanded medially, reduced posteriorly. Fan truncate, barely projected beyond posterior shield corners (Fig. 18A, D), margin smooth, with median notch.

Marginal shield chaetal fascicles include 11 lateral ones, chaetae of each fascicle in oval arrangement, and 10 posterior fascicles, chaetae in oval arrangement. The 11th lateral fascicles include one or two fine capillary chaetae, four times as long as others. Posterior fascicles positioned close to midline. Peg chaetae not visible, nor additional delicate capillary chaetae between lateral and posterior fascicles.

Branchiae numerous, not emerging from a distinct plate but from body wall; branchial area covered with thin, long interbranchial papillae, increasing in density towards margin of ventro-caudal shield (Fig. 18E).

##### Remarks.

*Petersenaspis capillata* (Nonato, 1966) comb. n. resembles *Petersenaspis palpallatoci* sp. n. because their shields have abundant long papillae, poorly defined ribs and no concentric lines. The main difference between them is the relative shield shape. In *Petersenaspis capillata* the anterior margin is barely projected forward and the posterior margin has a median notch, but no lateral notches, whereas in *Petersenaspis palpallatoci* the anterior margins are more projected forward and its posterior margin has a shallow median notch, plus two lateral notches. Other differences in the relative number of shield chaetal fascicles are less reliable because of chaetal fragility.

##### Distribution.

Only known from Central and Southern Brazilian localities, in 52–138 m depth. Omena and Amaral (1997) recorded this species from intertidal areas as well.

#### Petersenaspis palpallatoci sp. n.

urn:lsid:zoobank.org:act:30F5116A-B45D-40DA-B150-F4584F2E365D

http://species-id.net/wiki/Petersenaspis_palpallatoci

Figure 19

##### Type material.

**Philippine Islands, Sibuyan Sea**. Holotype (MNHN 1551) and paratype (MNHN 1552), MUSORSTROM, Cruise 3, Philippines, Sta. 140 (11°42.6'N, 122°31.5'E), E off Kalibo, 93 m, 6-VI-1985 (paratype with introvert invaginated).

**Additional material**. **Malasya.** 1 spec. (AM W196245), Sarawak, Bintulu, Similajan National Park, Sta 6, 5.5 m, 1982.

##### Description.

**Figure 19.**
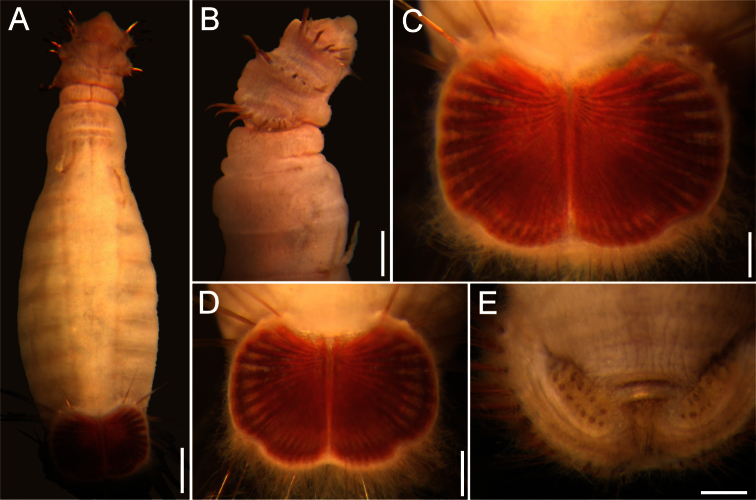
*Petersenaspis palpallatoci* sp. n. **A** Holotype (MNHN 1551), ventral view **B** Same, anterior end, lateral view **C** Same, ventro-caudal shield, frontal view **D** Paratype (MNHN 1552), ventro-caudal shield, frontal view **E** Same, branchial plate, frontal view, branchiae and papillae removed. Bars: **A** 1 mm **B** 0.7mm **C** 0.3 mm **D** 0.5 mm **E** 0.4 mm.

Holotype (MNHN 1551) with body pinkish anteriorly, whitish medially and posteriorly, clean with sparse, small filamentous papillae covering most of body (Fig. 19A). Larger, thin papillae along the dorsal surface of last few segments and surrounding shield but not arranged in rows. Body 11 mm long, 3 mm wide, 32 segments.

Prostomium projected, blunt conical (Fig. 19A, B). Peristomium rounded, equalized to the position of mouth, with abundant papillae extended behind prostomium. Mouth circular, extends from base of prostomium to anterior edge of first chaetiger.

First three chaetigers with 12–14 bright bronze recurved, spatulate hooks, without subdistal dark areas (Fig. 19B). Genital papillae protrude ventrally from body wall between segments 7 and 8. Pre-shield region with 8 segments, with single lateral bundles of about 2 capillary chaetae, protruding from body wall along segments 9–10.

Ventro-caudal shield brick red, papillose, with ribs faintly defined but no concentric lines, nor sediment particles; suture extended throughout shield. Anterior margins rounded; anterior depression shallow; anterior keels not exposed. Lateral margins rounded, expanded medially, reduced posteriorly. Fan truncate, barely projected beyond posterior shield corners (Fig. 19A, C), margin smooth, with a median and two smaller lateral notches.

Marginal shield chaetal fascicles include 10 lateral ones, chaetae in oval arrangement, and 10 posterior fascicles, chaetae in oval arrangement. The 11th lateral fascicles include one or two fine capillary chaetae, four times as long as others. Posterior fascicles positioned close to midline. Peg chaetae broken; additional delicate capillary chaetae between lateral and posterior fascicles present.

Branchiae few, emerging from a distinct plate; branchial area (observed in paratype) covered with very thin, long interbranchial papillae, increasing in density towards margin of ventro-caudal shield (Fig. 19E).

##### Variation.

Both the paratype and additional specimen have their introvert invaginated. Their shields show progressive enlargements of the anterior margins and the fan, with the median and lateral notches becoming more pronounced (Fig. 19D), and the shield taking a more elongate outline.

##### Etymology.

This species name is after Virgilio S. Palpal-latoc, researcher of the National Museum, Manila, in recognition of his many publications on the polychaete fauna of the Philippine Islands. The epithet is a noun in the genitive case.

##### Remarks.

*Petersenaspis palpallatoci* sp. n. resembles *Petersenaspis capillata* (Nonato, 1966) because both have shields with abundant long papillae, poorly defined ribs and no concentric lines. They differ in the shape of their shields. In *Petersenaspis palpallatoci* the anterior margins are more projected forward and its posterior margin has a shallow median notch plus two lateral notches, whereas in *Petersenaspis capillata* the anterior margin is barely projected forward and the posterior margin has a median notch, but no lateral notches. There are other differences regarding the relative number of shield chaetal fascicles, but because of chaetal fragility, they are less reliable.

##### Distribution.

Philippine Islands to Malasya, in 5.5–93 m depth.

#### Petersenaspis sp.

Figure 20

Sternaspis sp: Caullery 1944: 70.

##### Material examined.

**Indonesia, Lesser Sunda Islands**. 2 spec. (ZMA 1717), RV Siboga Exped., Sta. 300 (10°48.6'S, 123°23.1'E), 918 m, 30-I-1900. **Philippines.** 1 spec. (MNHN Musorstrom 3-94), Sta. 94 (13°47.4'S, 120°03.4'E), 780 m, 1-VI-1985.

##### Observations.

**Figure 20.**
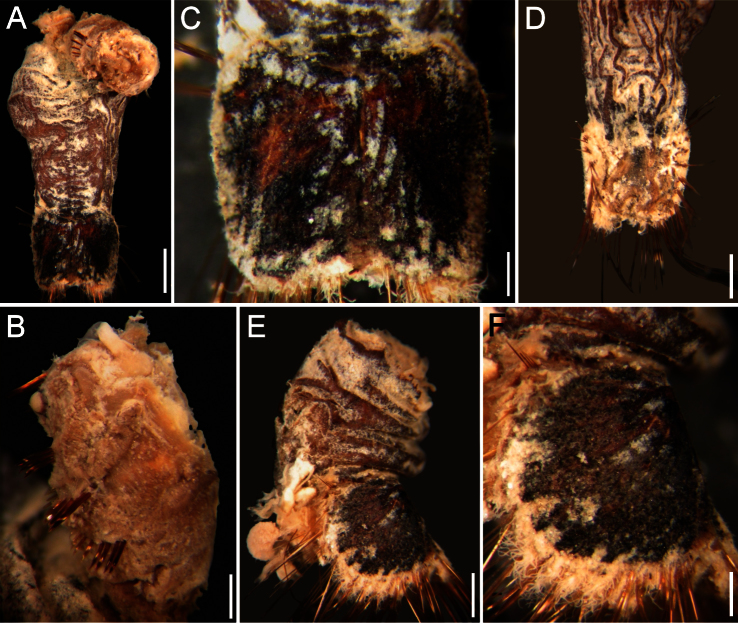
*Petersenaspis* sp (ZMA 1717) **A** Complete specimen, ventral view **B** Same, anterior segments in lateral view **C** Same, ventro-caudal shield, frontal view, lateral plates forced dorsally **D** Same, posterior region, dorsal view **E** Incomplete specimen, lateral view **F** Same, enlargement of right lateral shield plate. Bars: **A** 1.2 mm **B** 0.44 mm **C** 0.38 mm **D** 1 mm **E** 0.9 mm **F** 0.42 mm.

Two specimens (ZMA 1717), dried out. Longer, complete specimen (Fig. 20A) with introvert exposed, body wall breaking apart, 10 mm long, 3.3 mm wide. First three chaetigers with 10–12 bronze, subdistally expanded hooks (Fig. 20B). Ventro-caudal shield without sediment particles, longer than wide; anterior margins rounded, anterior depression shallow, lateral margins rounded (Fig. 20C); suture barely visible. Fan expanded beyond posterior corners, with median notch. Posterior region without branchiae, branchial plates not visible (Fig. 20D).

Smaller specimen (Fig. 20E) with introvert invaginated or broken off; body 5.8 mm long, 2.7 mm wide. Right ventro-caudal shield plate (Fig. 20F) with anterior and lateral margins rounded, fan with a median notch, with a smooth margin.

The other specimen (MNHN Musorstrom 3-94) with introvert invaginated; body 8 mm long, 5.5 mm wide. Ventro-caudal shield reddish with barely defined ribs and sediment particles removable by brushing. Chaetal fascicles better developed laterally, 10 bundles per side, and 6 posterior fascicles feebly developed per side.Branchiae lost, branchial plates barely projected, with abundant sediment particles.

##### Remarks.

Caullery (1944: 70) found two deep water specimens which were already corrugated and partly dehydrated when he observed them. The third specimen does not have the introvert exposed and the body wall is broken. These specimens belong to *Petersenaspis* because of several features: 1) the shield does not have well developed ribs, nor concentric lines on the surface, and 2) one of the Leiden specimens has spatulate introvert hooks. However, because of the state of the specimens, their complete description and affinities must wait until additional material becomes available. It can be indicated, however, that because of their shield shape, they resemble *Petersenaspis capillata* more than *Petersenaspis palpallatoci*, but because the Brazilian species was found in shallow water we think this is a different species.

##### Distribution.

Indonesia to the Philippine islands, 840–918 m depth.

## Supplementary Material

XML Treatment for Sternaspidae

XML Treatment for Sternaspis

XML Treatment for Sternaspis thalassemoides

XML Treatment for Sternaspis affinis

XML Treatment for Sternaspis africana

XML Treatment for Sternaspis andamanensis

XML Treatment for Sternaspis costata

XML Treatment for Sternaspis fossor

XML Treatment for Sternaspis islandica

XML Treatment for Sternaspis maior

XML Treatment for Sternaspis princeps

XML Treatment for Sternaspis rietschi

XML Treatment for Sternaspis scutata

XML Treatment for Sternaspis spinosa

XML Treatment for Sternaspis thorsoni

XML Treatment for Caulleryaspis

XML Treatment for Caulleryaspis gudmundssoni

XML Treatment for Caulleryaspis laevis

XML Treatment for Petersenaspis

XML Treatment for Petersenaspis capillata

XML Treatment for Petersenaspis palpallatoci

XML Treatment for Petersenaspis
